# Supplementary material for: Deciphering RTK-RAS and MAPK Pathway Dependencies in Gemcitabine-Treated Pancreatic Ductal Adenocarcinoma Through Conversational Artificial Intelligence
Source: Int J Mol Sci. 2026 Mar 26;27(7):3011. doi: 10.3390/ijms27073011 (PMC13072942; doi:10.3390/ijms27073011)
Supplement: Supplementary file 1 [file ijms-27-03011-s001.zip › ijms-4209837-supplementary.pdf]

Supplementary Materials:

Table S1. Comparison of Early-Onset PDAC Patients Treated with Gemcitabine Versus Those Not Treated with Gemcitabine.

RTK/RAS Pathway			
Gene	Early-Onset<br>Treated with Gemcitabine<br>n (%)	Early-Onset<br>Not Treated with Gemcitabine<br>n (%)	p-value
EGFR Mutation			
Present	0 (0.0%)	0 (0.0%)	1
Absent	15 (100.0%)	5 (100.0%)	
ERBB2 Mutation			
Present	0 (0.0%)	0 (0.0%)	1
Absent	15 (100.0%)	5 (100.0%)	
ERBB3 Mutation			
Present	0 (0.0%)	0 (0.0%)	1
Absent	15 (100.0%)	5 (100.0%)	
ERBB4 Mutation			
Present	0 (0.0%)	0 (0.0%)	1
Absent	15 (100.0%)	5 (100.0%)	
FGFR1 Mutation			
Present	0 (0.0%)	0 (0.0%)	1
Absent	15 (100.0%)	5 (100.0%)	
FGFR2 Mutation			
Present	0 (0.0%)	0 (0.0%)	1
Absent	15 (100.0%)	5 (100.0%)	
FGFR3 Mutation			
Present	0 (0.0%)	0 (0.0%)	1
Absent	15 (100.0%)	5 (100.0%)	
FGFR4 Mutation			
Present	0 (0.0%)	0 (0.0%)	1
Absent	15 (100.0%)	5 (100.0%)	
KRAS Mutation			
Present	11 (73.3%)	4 (80.0%)	1
Absent	4 (26.7%)	1 (20.0%)	
NRAS Mutation			
Present	0 (0.0%)	0 (0.0%)	1
Absent	15 (100.0%)	5 (100.0%)	
HRAS Mutation			
Present	0 (0.0%)	0 (0.0%)	1
Absent	15 (100.0%)	5 (100.0%)	

**BRAF Mutation**			
Present	0 (0.0%)	0 (0.0%)	1
Absent	15 (100.0%)	5 (100.0%)	
**MAP2K1 Mutation**			
Present	0 (0.0%)	0 (0.0%)	1
Absent	15 (100.0%)	5 (100.0%)	
**MAP2K2 Mutation**			
Present	0 (0.0%)	0 (0.0%)	1
Absent	15 (100.0%)	5 (100.0%)	
**MAPK1 Mutation**			
Present	0 (0.0%)	0 (0.0%)	1
Absent	15 (100.0%)	5 (100.0%)	
**MAPK3 Mutation**			
Present	0 (0.0%)	0 (0.0%)	1
Absent	15 (100.0%)	5 (100.0%)	
**SOS1 Mutation**			
Present	0 (0.0%)	0 (0.0%)	1
Absent	15 (100.0%)	5 (100.0%)	
**MET Mutation**			
Present	0 (0.0%)	0 (0.0%)	1
Absent	15 (100.0%)	5 (100.0%)	
**PDGFRA Mutation**			
Present	0 (0.0%)	0 (0.0%)	1
Absent	15 (100.0%)	5 (100.0%)	
**KIT Mutation**			
Present	0 (0.0%)	0 (0.0%)	1
Absent	15 (100.0%)	5 (100.0%)	
**IGF1R Mutation**			
Present	0 (0.0%)	0 (0.0%)	1
Absent	15 (100.0%)	5 (100.0%)	
**RET Mutation**			
Present	0 (0.0%)	0 (0.0%)	1
Absent	15 (100.0%)	5 (100.0%)	
**ROS1 Mutation**			
Present	0 (0.0%)	0 (0.0%)	1
Absent	15 (100.0%)	5 (100.0%)	
**ALK Mutation**			
Present	0 (0.0%)	0 (0.0%)	1
Absent	15 (100.0%)	5 (100.0%)	
**FLT3 Mutation**			

Present	0 (0.0%)	0 (0.0%)	1
Absent	15 (100.0%)	5 (100.0%)	
NTRK1 Mutation			
Present	1 (6.7%)	0 (0.0%)	1
Absent	14 (93.3%)	5 (100.0%)	
NTRK2 Mutation			
Present	0 (0.0%)	0 (0.0%)	1
Absent	15 (100.0%)	5 (100.0%)	
CBL Mutation			
Present	1 (6.7%)	0 (0.0%)	1
Absent	14 (93.3%)	5 (100.0%)	
ERRFI1 Mutation			
Present	0 (0.0%)	0 (0.0%)	1
Absent	15 (100.0%)	5 (100.0%)	
NF1 Mutation			
Present	0 (0.0%)	0 (0.0%)	1
Absent	15 (100.0%)	5 (100.0%)	
RASA1 Mutation			
Present	0 (0.0%)	0 (0.0%)	1
Absent	15 (100.0%)	5 (100.0%)	
PTPN11 Mutation			
Present	0 (0.0%)	0 (0.0%)	1
Absent	15 (100.0%)	5 (100.0%)	
RIT1 Mutation			
Present	0 (0.0%)	0 (0.0%)	1
Absent	15 (100.0%)	5 (100.0%)	
ARAF Mutation			
Present	0 (0.0%)	0 (0.0%)	1
Absent	15 (100.0%)	5 (100.0%)	
RAF1 Mutation			
Present	0 (0.0%)	0 (0.0%)	1
Absent	15 (100.0%)	5 (100.0%)	
RAC1 Mutation			
Present	0 (0.0%)	0 (0.0%)	1
Absent	15 (100.0%)	5 (100.0%)	

Table S2. Comparison of Late-Onset PDAC Patients Treated with Gemcitabine Versus Those Not Treated with Gemcitabine.

RTK/RAS Pathway

Gene	Late-Onset Treated with Gemcitabine n (%)	Late-Onset Not Treated with Gemcitabine n (%)	p-value
EGFR Mutation			
Present	2 (2.2%)	0 (0.0%)	0.503
Absent	89 (97.8%)	73 (100.0%)	
ERBB2 Mutation			
Present	6 (6.6%)	0 (0.0%)	**0.034**
Absent	85 (93.4%)	73 (100.0%)	
ERBB3 Mutation			
Present	2 (2.2%)	0 (0.0%)	0.503
Absent	89 (97.8%)	73 (100.0%)	
ERBB4 Mutation			
Present	5 (5.5%)	1 (1.4%)	0.2273
Absent	86 (94.5%)	72 (98.6%)	
FGFR1 Mutation			
Present	0 (0.0%)	0 (0.0%)	1
Absent	91 (100.0%)	73 (100.0%)	
FGFR2 Mutation			
Present	0 (0.0%)	0 (0.0%)	1
Absent	91 (100.0%)	73 (100.0%)	
FGFR3 Mutation			
Present	2 (2.2%)	0 (0.0%)	0.503
Absent	89 (97.8%)	73 (100.0%)	
FGFR4 Mutation			
Present	1 (1.1%)	0 (0.0%)	1
Absent	90 (98.9%)	73 (100.0%)	
KRAS Mutation			
Present	60 (65.9%)	46 (63.0%)	0.8224
Absent	31 (34.1%)	27 (37.0%)	
NRAS Mutation			
Present	0 (0.0%)	0 (0.0%)	1
Absent	91 (100.0%)	73 (100.0%)	
HRAS Mutation			
Present	1 (1.1%)	0 (0.0%)	1
Absent	90 (98.9%)	73 (100.0%)	
BRAF Mutation			
Present	0 (0.0%)	2 (2.7%)	0.1966
Absent	91 (100.0%)	71 (97.3%)	
MAP2K1 Mutation			

Present	0 (0.0%)	0 (0.0%)	1
Absent	91 (100.0%)	73 (100.0%)	
**MAP2K2 Mutation**			
Present	0 (0.0%)	0 (0.0%)	1
Absent	91 (100.0%)	73 (100.0%)	
**MAPK1 Mutation**			
Present	0 (0.0%)	0 (0.0%)	1
Absent	91 (100.0%)	73 (100.0%)	
**MAPK3 Mutation**			
Present	0 (0.0%)	0 (0.0%)	1
Absent	91 (100.0%)	73 (100.0%)	
**SOS1 Mutation**			
Present	2 (2.2%)	0 (0.0%)	0.503
Absent	89 (97.8%)	73 (100.0%)	
**MET Mutation**			
Present	4 (4.4%)	0 (0.0%)	0.1295
Absent	87 (95.6%)	73 (100.0%)	
**PDGFRA Mutation**			
Present	2 (2.2%)	0 (0.0%)	0.503
Absent	89 (97.8%)	73 (100.0%)	
**KIT Mutation**			
Present	3 (3.3%)	0 (0.0%)	0.2545
Absent	88 (96.7%)	73 (100.0%)	
**IGF1R Mutation**			
Present	3 (3.3%)	0 (0.0%)	0.2545
Absent	88 (96.7%)	73 (100.0%)	
**RET Mutation**			
Present	7 (7.7%)	0 (0.0%)	**0.0175**
Absent	84 (92.3%)	73 (100.0%)	
**ROS1 Mutation**			
Present	4 (4.4%)	0 (0.0%)	0.1295
Absent	87 (95.6%)	73 (100.0%)	
**ALK Mutation**			
Present	2 (2.2%)	0 (0.0%)	0.503
Absent	89 (97.8%)	73 (100.0%)	
**FLT3 Mutation**			
Present	2 (2.2%)	1 (1.4%)	1
Absent	89 (97.8%)	72 (98.6%)	
**NTRK1 Mutation**			
Present	1 (1.1%)	1 (1.4%)	1

Absent	90 (98.9%)	72 (98.6%)	
**NTRK2 Mutation**			
Present	2 (2.2%)	0 (0.0%)	0.503
Absent	89 (97.8%)	73 (100.0%)	
**CBL Mutation**			
Present	0 (0.0%)	0 (0.0%)	1
Absent	91 (100.0%)	73 (100.0%)	
**ERRFI1 Mutation**			
Present	0 (0.0%)	0 (0.0%)	1
Absent	91 (100.0%)	73 (100.0%)	
**NF1 Mutation**			
Present	2 (2.2%)	0 (0.0%)	0.503
Absent	89 (97.8%)	73 (100.0%)	
**RASA1 Mutation**			
Present	1 (1.1%)	1 (1.4%)	1
Absent	90 (98.9%)	72 (98.6%)	
**PTPN11 Mutation**			
Present	0 (0.0%)	0 (0.0%)	1
Absent	91 (100.0%)	73 (100.0%)	
**RIT1 Mutation**			
Present	0 (0.0%)	0 (0.0%)	1
Absent	91 (100.0%)	73 (100.0%)	
**ARAF Mutation**			
Present	3 (3.3%)	0 (0.0%)	0.2545
Absent	88 (96.7%)	73 (100.0%)	
**RAF1 Mutation**			
Present	0 (0.0%)	0 (0.0%)	1
Absent	91 (100.0%)	73 (100.0%)	
**RAC1 Mutation**			
Present	0 (0.0%)	0 (0.0%)	1
Absent	91 (100.0%)	73 (100.0%)	

Table S3. Comparison of Early-Onset PDAC Patients Versus Late-Onset PDAC Patients Treated with Gemcitabine.

RTK/RAS Pathway			
Gene	Early-Onset<br>Treated with Gemcitabine<br>n (%)	Late-Onset<br>Treated with Gemcitabine<br>n (%)	p-value
**EGFR Mutation**			
Present	0 (0.0%)	2 (2.2%)	1
Absent	15 (100.0%)	89 (97.8%)	

ERBB2 Mutation			
Present	0 (0.0%)	6 (6.6%)	0.5911
Absent	15 (100.0%)	85 (93.4%)	
ERBB3 Mutation			
Present	0 (0.0%)	2 (2.2%)	1
Absent	15 (100.0%)	89 (97.8%)	
ERBB4 Mutation			
Present	0 (0.0%)	5 (5.5%)	1
Absent	15 (100.0%)	86 (94.5%)	
FGFR1 Mutation			
Present	0 (0.0%)	0 (0.0%)	1
Absent	15 (100.0%)	91 (100.0%)	
FGFR2 Mutation			
Present	0 (0.0%)	0 (0.0%)	1
Absent	15 (100.0%)	91 (100.0%)	
FGFR3 Mutation			
Present	0 (0.0%)	2 (2.2%)	1
Absent	15 (100.0%)	89 (97.8%)	
FGFR4 Mutation			
Present	0 (0.0%)	1 (1.1%)	1
Absent	15 (100.0%)	90 (98.9%)	
KRAS Mutation			
Present	11 (73.3%)	60 (65.9%)	0.7688
Absent	4 (26.7%)	31 (34.1%)	
NRAS Mutation			
Present	0 (0.0%)	0 (0.0%)	1
Absent	15 (100.0%)	91 (100.0%)	
HRAS Mutation			
Present	0 (0.0%)	1 (1.1%)	1
Absent	15 (100.0%)	90 (98.9%)	
BRAF Mutation			
Present	0 (0.0%)	0 (0.0%)	1
Absent	15 (100.0%)	91 (100.0%)	
MAP2K1 Mutation			
Present	0 (0.0%)	0 (0.0%)	1
Absent	15 (100.0%)	91 (100.0%)	
MAP2K2 Mutation			
Present	0 (0.0%)	0 (0.0%)	1
Absent	15 (100.0%)	91 (100.0%)	
MAPK1 Mutation			

Present	0 (0.0%)	0 (0.0%)	1
Absent	15 (100.0%)	91 (100.0%)	
**MAPK3 Mutation**			
Present	0 (0.0%)	0 (0.0%)	1
Absent	15 (100.0%)	91 (100.0%)	
**SOS1 Mutation**			
Present	0 (0.0%)	2 (2.2%)	1
Absent	15 (100.0%)	89 (97.8%)	
**MET Mutation**			
Present	0 (0.0%)	4 (4.4%)	1
Absent	15 (100.0%)	87 (95.6%)	
**PDGFRA Mutation**			
Present	0 (0.0%)	2 (2.2%)	1
Absent	15 (100.0%)	89 (97.8%)	
**KIT Mutation**			
Present	0 (0.0%)	3 (3.3%)	1
Absent	15 (100.0%)	88 (96.7%)	
**IGF1R Mutation**			
Present	0 (0.0%)	3 (3.3%)	1
Absent	15 (100.0%)	88 (96.7%)	
**RET Mutation**			
Present	0 (0.0%)	7 (7.7%)	0.5897
Absent	15 (100.0%)	84 (92.3%)	
**ROS1 Mutation**			
Present	0 (0.0%)	4 (4.4%)	1
Absent	15 (100.0%)	87 (95.6%)	
**ALK Mutation**			
Present	0 (0.0%)	2 (2.2%)	1
Absent	15 (100.0%)	89 (97.8%)	
**FLT3 Mutation**			
Present	0 (0.0%)	2 (2.2%)	1
Absent	15 (100.0%)	89 (97.8%)	
**NTRK1 Mutation**			
Present	1 (6.7%)	1 (1.1%)	0.2642
Absent	14 (93.3%)	90 (98.9%)	
**NTRK2 Mutation**			
Present	0 (0.0%)	2 (2.2%)	1
Absent	15 (100.0%)	89 (97.8%)	
**CBL Mutation**			
Present	1 (6.7%)	0 (0.0%)	0.1415

Absent	14 (93.3%)	91 (100.0%)	
**ERRFI1 Mutation**			
Present	0 (0.0%)	0 (0.0%)	1
Absent	15 (100.0%)	91 (100.0%)	
**NF1 Mutation**			
Present	0 (0.0%)	2 (2.2%)	1
Absent	15 (100.0%)	89 (97.8%)	
**RASA1 Mutation**			
Present	0 (0.0%)	1 (1.1%)	1
Absent	15 (100.0%)	90 (98.9%)	
**PTPN11 Mutation**			
Present	0 (0.0%)	0 (0.0%)	1
Absent	15 (100.0%)	91 (100.0%)	
**RIT1 Mutation**			
Present	0 (0.0%)	0 (0.0%)	1
Absent	15 (100.0%)	91 (100.0%)	
**ARAF Mutation**			
Present	0 (0.0%)	3 (3.3%)	1
Absent	15 (100.0%)	88 (96.7%)	
**RAF1 Mutation**			
Present	0 (0.0%)	0 (0.0%)	1
Absent	15 (100.0%)	91 (100.0%)	
**RAC1 Mutation**			
Present	0 (0.0%)	0 (0.0%)	1
Absent	15 (100.0%)	91 (100.0%)	

Table S4. Comparison of Early-Onset PDAC Patients Versus Late-Onset PDAC Patients Not Treated with Gemcitabine.

RTK/RAS Pathway			
Gene	Early-Onset Not Treated with Gemcitabine n (%)	Late-Onset Not Treated with Gemcitabine n (%)	p-value
**EGFR Mutation**			
Present	0 (0.0%)	0 (0.0%)	1
Absent	5 (100.0%)	73 (100.0%)	
**ERBB2 Mutation**			
Present	0 (0.0%)	0 (0.0%)	1
Absent	5 (100.0%)	73 (100.0%)	
**ERBB3 Mutation**			
Present	0 (0.0%)	0 (0.0%)	1
Absent	5 (100.0%)	73 (100.0%)	

**ERBB4 Mutation**			
Present	0 (0.0%)	1 (1.4%)	1
Absent	5 (100.0%)	72 (98.6%)	
**FGFR1 Mutation**			
Present	0 (0.0%)	0 (0.0%)	1
Absent	5 (100.0%)	73 (100.0%)	
**FGFR2 Mutation**			
Present	0 (0.0%)	0 (0.0%)	1
Absent	5 (100.0%)	73 (100.0%)	
**FGFR3 Mutation**			
Present	0 (0.0%)	0 (0.0%)	1
Absent	5 (100.0%)	73 (100.0%)	
**FGFR4 Mutation**			
Present	0 (0.0%)	0 (0.0%)	1
Absent	5 (100.0%)	73 (100.0%)	
**KRAS Mutation**			
Present	4 (80.0%)	46 (63.0%)	0.6491
Absent	1 (20.0%)	27 (37.0%)	
**NRAS Mutation**			
Present	0 (0.0%)	0 (0.0%)	1
Absent	5 (100.0%)	73 (100.0%)	
**HRAS Mutation**			
Present	0 (0.0%)	0 (0.0%)	1
Absent	5 (100.0%)	73 (100.0%)	
**BRAF Mutation**			
Present	0 (0.0%)	2 (2.7%)	1
Absent	5 (100.0%)	71 (97.3%)	
**MAP2K1 Mutation**			
Present	0 (0.0%)	0 (0.0%)	1
Absent	5 (100.0%)	73 (100.0%)	
**MAP2K2 Mutation**			
Present	0 (0.0%)	0 (0.0%)	1
Absent	5 (100.0%)	73 (100.0%)	
**MAPK1 Mutation**			
Present	0 (0.0%)	0 (0.0%)	1
Absent	5 (100.0%)	73 (100.0%)	
**MAPK3 Mutation**			
Present	0 (0.0%)	0 (0.0%)	1
Absent	5 (100.0%)	73 (100.0%)	
**SOS1 Mutation**			

Present	0 (0.0%)	0 (0.0%)	1
Absent	5 (100.0%)	73 (100.0%)	
MET Mutation			
Present	0 (0.0%)	0 (0.0%)	1
Absent	5 (100.0%)	73 (100.0%)	
PDGFRA Mutation			
Present	0 (0.0%)	0 (0.0%)	1
Absent	5 (100.0%)	73 (100.0%)	
KIT Mutation			
Present	0 (0.0%)	0 (0.0%)	1
Absent	5 (100.0%)	73 (100.0%)	
IGF1R Mutation			
Present	0 (0.0%)	0 (0.0%)	1
Absent	5 (100.0%)	73 (100.0%)	
RET Mutation			
Present	0 (0.0%)	0 (0.0%)	1
Absent	5 (100.0%)	73 (100.0%)	
ROS1 Mutation			
Present	0 (0.0%)	0 (0.0%)	1
Absent	5 (100.0%)	73 (100.0%)	
ALK Mutation			
Present	0 (0.0%)	0 (0.0%)	1
Absent	5 (100.0%)	73 (100.0%)	
FLT3 Mutation			
Present	0 (0.0%)	1 (1.4%)	1
Absent	5 (100.0%)	72 (98.6%)	
NTRK1 Mutation			
Present	0 (0.0%)	1 (1.4%)	1
Absent	5 (100.0%)	72 (98.6%)	
NTRK2 Mutation			
Present	0 (0.0%)	0 (0.0%)	1
Absent	5 (100.0%)	73 (100.0%)	
CBL Mutation			
Present	0 (0.0%)	0 (0.0%)	1
Absent	5 (100.0%)	73 (100.0%)	
ERRFI1 Mutation			
Present	0 (0.0%)	0 (0.0%)	1
Absent	5 (100.0%)	73 (100.0%)	
NF1 Mutation			
Present	0 (0.0%)	0 (0.0%)	1

Absent	5 (100.0%)	73 (100.0%)	
**RASA1 Mutation**			
Present	0 (0.0%)	1 (1.4%)	1
Absent	5 (100.0%)	72 (98.6%)	
**PTPN11 Mutation**			
Present	0 (0.0%)	0 (0.0%)	1
Absent	5 (100.0%)	73 (100.0%)	
**RIT1 Mutation**			
Present	0 (0.0%)	0 (0.0%)	1
Absent	5 (100.0%)	73 (100.0%)	
**ARAF Mutation**			
Present	0 (0.0%)	0 (0.0%)	1
Absent	5 (100.0%)	73 (100.0%)	
**RAF1 Mutation**			
Present	0 (0.0%)	0 (0.0%)	1
Absent	5 (100.0%)	73 (100.0%)	
**RAC1 Mutation**			
Present	0 (0.0%)	0 (0.0%)	1
Absent	5 (100.0%)	73 (100.0%)	

Table S5. Comparison of Early-Onset PDAC Patients Treated with Gemcitabine Versus Those Not Treated with Gemcitabine.

MAPK Pathway			
Gene	Early-Onset Treated with Gemcitabine n (%)	Early-Onset Not Treated with Gemcitabine n (%)	p-value
**ACVR1 Mutation**			
Present	0 (0.0%)	0 (0.0%)	1
Absent	15 (100.0%)	5 (100.0%)	
**AKT1 Mutation**			
Present	0 (0.0%)	0 (0.0%)	1
Absent	15 (100.0%)	5 (100.0%)	
**AKT2 Mutation**			
Present	0 (0.0%)	0 (0.0%)	1
Absent	15 (100.0%)	5 (100.0%)	
**AKT3 Mutation**			
Present	0 (0.0%)	0 (0.0%)	1
Absent	15 (100.0%)	5 (100.0%)	
**BRAF Mutation**			
Present	0 (0.0%)	0 (0.0%)	1
Absent	15 (100.0%)	5 (100.0%)	

CACNA1A Mutation			
Present	1 (6.7%)	0 (0.0%)	1
Absent	14 (93.3%)	5 (100.0%)	
CACNA1B Mutation			
Present	0 (0.0%)	0 (0.0%)	1
Absent	15 (100.0%)	5 (100.0%)	
CACNA1C Mutation			
Present	0 (0.0%)	0 (0.0%)	1
Absent	15 (100.0%)	5 (100.0%)	
CACNA1D Mutation			
Present	1 (6.7%)	0 (0.0%)	1
Absent	14 (93.3%)	5 (100.0%)	
CACNA1E Mutation			
Present	0 (0.0%)	0 (0.0%)	1
Absent	15 (100.0%)	5 (100.0%)	
CACNA1F Mutation			
Present	0 (0.0%)	0 (0.0%)	1
Absent	15 (100.0%)	5 (100.0%)	
CACNA1G Mutation			
Present	1 (6.7%)	0 (0.0%)	1
Absent	14 (93.3%)	5 (100.0%)	
CACNA1H Mutation			
Present	0 (0.0%)	0 (0.0%)	1
Absent	15 (100.0%)	5 (100.0%)	
CACNA1I Mutation			
Present	0 (0.0%)	0 (0.0%)	1
Absent	15 (100.0%)	5 (100.0%)	
CACNA1S Mutation			
Present	0 (0.0%)	0 (0.0%)	1
Absent	15 (100.0%)	5 (100.0%)	
CACNA2D1 Mutation			
Present	0 (0.0%)	2 (40.0%)	*0.0526*
Absent	15 (100.0%)	3 (60.0%)	
CACNA2D3 Mutation			
Present	0 (0.0%)	2 (40.0%)	*0.0526*
Absent	15 (100.0%)	3 (60.0%)	
CACNA2D4 Mutation			
Present	0 (0.0%)	2 (40.0%)	*0.0526*
Absent	15 (100.0%)	3 (60.0%)	
CACNB1 Mutation			

Present	0 (0.0%)	0 (0.0%)	1
Absent	15 (100.0%)	5 (100.0%)	
**CACNB2 Mutation**			
Present	0 (0.0%)	0 (0.0%)	1
Absent	15 (100.0%)	5 (100.0%)	
**CACNG1 Mutation**			
Present	0 (0.0%)	0 (0.0%)	1
Absent	15 (100.0%)	5 (100.0%)	
**CACNG2 Mutation**			
Present	0 (0.0%)	0 (0.0%)	1
Absent	15 (100.0%)	5 (100.0%)	
**CACNG3 Mutation**			
Present	1 (6.7%)	0 (0.0%)	1
Absent	14 (93.3%)	5 (100.0%)	
**CACNB2 Mutation**			
Present	0 (0.0%)	0 (0.0%)	1
Absent	15 (100.0%)	5 (100.0%)	
**CDC42 Mutation**			
Present	0 (0.0%)	0 (0.0%)	1
Absent	15 (100.0%)	5 (100.0%)	
**CRK Mutation**			
Present	0 (0.0%)	0 (0.0%)	1
Absent	15 (100.0%)	5 (100.0%)	
**DAXX Mutation**			
Present	0 (0.0%)	0 (0.0%)	1
Absent	15 (100.0%)	5 (100.0%)	
**DUSP6 Mutation**			
Present	0 (0.0%)	0 (0.0%)	1
Absent	15 (100.0%)	5 (100.0%)	
**DUSP7 Mutation**			
Present	0 (0.0%)	0 (0.0%)	1
Absent	15 (100.0%)	5 (100.0%)	
**DUSP10 Mutation**			
Present	0 (0.0%)	0 (0.0%)	1
Absent	15 (100.0%)	5 (100.0%)	
**DUSP14 Mutation**			
Present	0 (0.0%)	0 (0.0%)	1
Absent	15 (100.0%)	5 (100.0%)	
**EGFR Mutation**			
Present	0 (0.0%)	0 (0.0%)	1

Absent	15 (100.0%)	5 (100.0%)	
**FGF12 Mutation**			
Present	0 (0.0%)	0 (0.0%)	1
Absent	15 (100.0%)	5 (100.0%)	
**FGF13 Mutation**			
Present	0 (0.0%)	0 (0.0%)	1
Absent	15 (100.0%)	5 (100.0%)	
**FGF14 Mutation**			
Present	0 (0.0%)	0 (0.0%)	1
Absent	15 (100.0%)	5 (100.0%)	
**FGF17 Mutation**			
Present	0 (0.0%)	0 (0.0%)	1
Absent	15 (100.0%)	5 (100.0%)	
**FGF2 Mutation**			
Present	0 (0.0%)	0 (0.0%)	1
Absent	15 (100.0%)	5 (100.0%)	
**FGF23 Mutation**			
Present	0 (0.0%)	0 (0.0%)	1
Absent	15 (100.0%)	5 (100.0%)	
**FGF6 Mutation**			
Present	0 (0.0%)	1 (20.0%)	0.25
Absent	15 (100.0%)	4 (80.0%)	
**FGFR3 Mutation**			
Present	0 (0.0%)	0 (0.0%)	1
Absent	15 (100.0%)	5 (100.0%)	
**FGFR4 Mutation**			
Present	0 (0.0%)	0 (0.0%)	1
Absent	15 (100.0%)	5 (100.0%)	
**FLNA Mutation**			
Present	2 (13.3%)	0 (0.0%)	1
Absent	13 (86.7%)	5 (100.0%)	
**FLNB Mutation**			
Present	2 (13.3%)	0 (0.0%)	1
Absent	13 (86.7%)	5 (100.0%)	
**FLNC Mutation**			
Present	1 (6.7%)	0 (0.0%)	1
Absent	14 (93.3%)	5 (100.0%)	
**HRAS Mutation**			
Present	0 (0.0%)	0 (0.0%)	1
Absent	15 (100.0%)	5 (100.0%)	

**JUND Mutation**			
Present	0 (0.0%)	0 (0.0%)	1
Absent	15 (100.0%)	5 (100.0%)	
**KRAS Mutation**			
Present	11 (73.3%)	4 (80.0%)	1
Absent	4 (26.7%)	1 (20.0%)	
**MAP2K4 Mutation**			
Present	0 (0.0%)	0 (0.0%)	1
Absent	15 (100.0%)	5 (100.0%)	
**MAP2K6 Mutation**			
Present	0 (0.0%)	0 (0.0%)	1
Absent	15 (100.0%)	5 (100.0%)	
**MAP2K7 Mutation**			
Present	0 (0.0%)	0 (0.0%)	1
Absent	15 (100.0%)	5 (100.0%)	
**MAP3K1 Mutation**			
Present	0 (0.0%)	0 (0.0%)	1
Absent	15 (100.0%)	5 (100.0%)	
**MAP3K10 Mutation**			
Present	0 (0.0%)	0 (0.0%)	1
Absent	15 (100.0%)	5 (100.0%)	
**MAP3K12 Mutation**			
Present	0 (0.0%)	0 (0.0%)	1
Absent	15 (100.0%)	5 (100.0%)	
**MAP3K13 Mutation**			
Present	0 (0.0%)	0 (0.0%)	1
Absent	15 (100.0%)	5 (100.0%)	
**MAP3K2 Mutation**			
Present	0 (0.0%)	0 (0.0%)	1
Absent	15 (100.0%)	5 (100.0%)	
**MAP3K4 Mutation**			
Present	0 (0.0%)	0 (0.0%)	1
Absent	15 (100.0%)	5 (100.0%)	
**MAP3K5 Mutation**			
Present	0 (0.0%)	0 (0.0%)	1
Absent	15 (100.0%)	5 (100.0%)	
**MAP3K6 Mutation**			
Present	0 (0.0%)	0 (0.0%)	1
Absent	15 (100.0%)	5 (100.0%)	
**MAP4K1 Mutation**			

Present	0 (0.0%)	0 (0.0%)	1
Absent	15 (100.0%)	5 (100.0%)	
**MAP4K2 Mutation**			
Present	0 (0.0%)	0 (0.0%)	1
Absent	15 (100.0%)	5 (100.0%)	
**MAP4K3 Mutation**			
Present	0 (0.0%)	0 (0.0%)	1
Absent	15 (100.0%)	5 (100.0%)	
**MAP4K4 Mutation**			
Present	0 (0.0%)	0 (0.0%)	1
Absent	15 (100.0%)	5 (100.0%)	
**MAPK10 Mutation**			
Present	0 (0.0%)	0 (0.0%)	1
Absent	15 (100.0%)	5 (100.0%)	
**MAPK11 Mutation**			
Present	0 (0.0%)	0 (0.0%)	1
Absent	15 (100.0%)	5 (100.0%)	
**MAPK12 Mutation**			
Present	0 (0.0%)	0 (0.0%)	1
Absent	15 (100.0%)	5 (100.0%)	
**MAPK13 Mutation**			
Present	0 (0.0%)	0 (0.0%)	1
Absent	15 (100.0%)	5 (100.0%)	
**MOS Mutation**			
Present	1 (6.7%)	0 (0.0%)	1
Absent	14 (93.3%)	5 (100.0%)	
**MYC Mutation**			
Present	0 (0.0%)	0 (0.0%)	1
Absent	15 (100.0%)	5 (100.0%)	
**NF1 Mutation**			
Present	0 (0.0%)	0 (0.0%)	1
Absent	15 (100.0%)	5 (100.0%)	
**NFATC2 Mutation**			
Present	1 (6.7%)	0 (0.0%)	1
Absent	14 (93.3%)	5 (100.0%)	
**NRAS Mutation**			
Present	0 (0.0%)	0 (0.0%)	1
Absent	15 (100.0%)	5 (100.0%)	
**NTRK1 Mutation**			
Present	1 (6.7%)	0 (0.0%)	0.4444

Absent	3 (20.0%)	5 (100.0%)	
**NTRK2 Mutation**			
Present	0 (0.0%)	0 (0.0%)	1
Absent	15 (100.0%)	5 (100.0%)	
**PAK2 Mutation**			
Present	0 (0.0%)	0 (0.0%)	1
Absent	15 (100.0%)	5 (100.0%)	
**PDGFRA Mutation**			
Present	0 (0.0%)	0 (0.0%)	1
Absent	15 (100.0%)	5 (100.0%)	
**PDGFRB Mutation**			
Present	0 (0.0%)	0 (0.0%)	1
Absent	15 (100.0%)	5 (100.0%)	
**PLA2G6 Mutation**			
Present	0 (0.0%)	0 (0.0%)	1
Absent	15 (100.0%)	5 (100.0%)	
**PRKACB Mutation**			
Present	1 (6.7%)	0 (0.0%)	1
Absent	14 (93.3%)	5 (100.0%)	
**RAC2 Mutation**			
Present	0 (0.0%)	0 (0.0%)	1
Absent	15 (100.0%)	5 (100.0%)	
**RAPGEF2 Mutation**			
Present	0 (0.0%)	0 (0.0%)	1
Absent	15 (100.0%)	5 (100.0%)	
**RASA1 Mutation**			
Present	0 (0.0%)	0 (0.0%)	1
Absent	15 (100.0%)	5 (100.0%)	
**RPS6KA4 Mutation**			
Present	0 (0.0%)	0 (0.0%)	1
Absent	15 (100.0%)	5 (100.0%)	
**RPS6KA6 Mutation**			
Present	0 (0.0%)	0 (0.0%)	1
Absent	15 (100.0%)	5 (100.0%)	
**SOS1 Mutation**			
Present	0 (0.0%)	0 (0.0%)	1
Absent	15 (100.0%)	5 (100.0%)	
**SOS2 Mutation**			
Present	0 (0.0%)	0 (0.0%)	1
Absent	15 (100.0%)	5 (100.0%)	

**TGFB1 Mutation**			
Present	0 (0.0%)	0 (0.0%)	1
Absent	15 (100.0%)	5 (100.0%)	
**TGFBR1 Mutation**			
Present	0 (0.0%)	0 (0.0%)	1
Absent	15 (100.0%)	5 (100.0%)	
**TGFBR2 Mutation**			
Present	1 (6.7%)	0 (0.0%)	1
Absent	14 (93.3%)	5 (100.0%)	
**TP53 Mutation**			
Present	13 (86.7%)	2 (40.0%)	0.0726
Absent	2 (13.3%)	3 (60.0%)	
**TRAF2 Mutation**			
Present	0 (0.0%)	0 (0.0%)	1
Absent	15 (100.0%)	5 (100.0%)	

Table S6. Comparison of Late-Onset PDAC Patients Treated with Gemcitabine Versus Those Not Treated with Gemcitabine.

MAPK Pathway			
Gene	Late-Onset<br>Treated with Gemcitabine<br>n (%)	Late-Onset<br>Not Treated with Gemcitabine<br>n (%)	p-value
**ACVR1 Mutation**			
Present	0 (0.0%)	0 (0.0%)	1
Absent	91 (100.0%)	73 (100.0%)	
**AKT1 Mutation**			
Present	0 (0.0%)	0 (0.0%)	1
Absent	91 (100.0%)	73 (100.0%)	
**AKT2 Mutation**			
Present	1 (1.1%)	0 (0.0%)	1
Absent	90 (98.9%)	73 (100.0%)	
**AKT3 Mutation**			
Present	1 (1.1%)	0 (0.0%)	1
Absent	90 (98.9%)	73 (100.0%)	
**BRAF Mutation**			
Present	0 (0.0%)	2 (2.7%)	0.1966
Absent	91 (100.0%)	71 (97.3%)	
**CACNA1A Mutation**			
Present	5 (5.5%)	1 (1.4%)	0.2273
Absent	86 (94.5%)	72 (98.6%)	
**CACNA1B Mutation**			

Present	7 (7.7%)	4 (5.5%)	0.7561
Absent	84 (92.3%)	69 (94.5%)	
**CACNA1C Mutation**			
Present	9 (9.9%)	0 (0.0%)	**0.0047**
Absent	82 (90.1%)	73 (100.0%)	
**CACNA1D Mutation**			
Present	5 (5.5%)	1 (1.4%)	0.2273
Absent	86 (94.5%)	72 (98.6%)	
**CACNA1E Mutation**			
Present	7 (7.7%)	0 (0.0%)	**0.0175**
Absent	84 (92.3%)	73 (100.0%)	
**CACNA1F Mutation**			
Present	1 (1.1%)	1 (1.4%)	1
Absent	90 (98.9%)	72 (98.6%)	
**CACNA1G Mutation**			
Present	5 (5.5%)	1 (1.4%)	0.2273
Absent	86 (94.5%)	72 (98.6%)	
**CACNA1H Mutation**			
Present	3 (3.3%)	1 (1.4%)	0.6296
Absent	88 (96.7%)	72 (98.6%)	
**CACNA1I Mutation**			
Present	4 (4.4%)	2 (2.7%)	0.6933
Absent	87 (95.6%)	71 (97.3%)	
**CACNA1S Mutation**			
Present	5 (5.5%)	1 (1.4%)	0.2273
Absent	86 (94.5%)	72 (98.6%)	
**CACNA2D1 Mutation**			
Present	0 (0.0%)	1 (1.4%)	0.4451
Absent	91 (100.0%)	72 (98.6%)	
**CACNA2D3 Mutation**			
Present	0 (0.0%)	1 (1.4%)	0.4451
Absent	91 (100.0%)	72 (98.6%)	
**CACNA2D4 Mutation**			
Present	0 (0.0%)	1 (1.4%)	0.4451
Absent	91 (100.0%)	72 (98.6%)	
**CACNB1 Mutation**			
Present	0 (0.0%)	2 (2.7%)	0.1966
Absent	91 (100.0%)	71 (97.3%)	
**CACNB2 Mutation**			
Present	0 (0.0%)	2 (2.7%)	0.1966

Absent	91 (100.0%)	71 (97.3%)	
**CACNG1 Mutation**			
Present	1 (1.1%)	0 (0.0%)	1
Absent	90 (98.9%)	73 (100.0%)	
**CACNG2 Mutation**			
Present	2 (2.2%)	0 (0.0%)	0.503
Absent	89 (97.8%)	73 (100.0%)	
**CACNG3 Mutation**			
Present	0 (0.0%)	1 (1.4%)	0.4451
Absent	91 (100.0%)	72 (98.6%)	
**CACNB2 Mutation**			
Present	1 (1.1%)	0 (0.0%)	1
Absent	90 (98.9%)	73 (100.0%)	
**CDC42 Mutation**			
Present	0 (0.0%)	1 (1.4%)	0.4451
Absent	91 (100.0%)	72 (98.6%)	
**CRK Mutation**			
Present	0 (0.0%)	0 (0.0%)	1
Absent	91 (100.0%)	73 (100.0%)	
**DAXX Mutation**			
Present	2 (2.2%)	1 (1.4%)	1
Absent	89 (97.8%)	72 (98.6%)	
**DUSP6 Mutation**			
Present	2 (2.2%)	0 (0.0%)	0.503
Absent	89 (97.8%)	73 (100.0%)	
**DUSP7 Mutation**			
Present	2 (2.2%)	0 (0.0%)	0.503
Absent	89 (97.8%)	73 (100.0%)	
**DUSP10 Mutation**			
Present	1 (1.1%)	0 (0.0%)	1
Absent	90 (98.9%)	73 (100.0%)	
**DUSP14 Mutation**			
Present	1 (1.1%)	0 (0.0%)	1
Absent	90 (98.9%)	73 (100.0%)	
**EGFR Mutation**			
Present	2 (2.2%)	0 (0.0%)	0.503
Absent	89 (97.8%)	73 (100.0%)	
**FGF12 Mutation**			
Present	1 (1.1%)	0 (0.0%)	1
Absent	90 (98.9%)	73 (100.0%)	

**FGF13 Mutation**			
Present	1 (1.1%)	1 (1.4%)	1
Absent	90 (98.9%)	72 (98.6%)	
**FGF14 Mutation**			
Present	2 (2.2%)	0 (0.0%)	0.503
Absent	89 (97.8%)	73 (100.0%)	
**FGF17 Mutation**			
Present	1 (1.1%)	0 (0.0%)	1
Absent	90 (98.9%)	73 (100.0%)	
**FGF2 Mutation**			
Present	1 (1.1%)	0 (0.0%)	1
Absent	90 (98.9%)	73 (100.0%)	
**FGF23 Mutation**			
Present	1 (1.1%)	0 (0.0%)	1
Absent	90 (98.9%)	73 (100.0%)	
**FGF6 Mutation**			
Present	1 (1.1%)	0 (0.0%)	1
Absent	90 (98.9%)	73 (100.0%)	
**FGFR3 Mutation**			
Present	2 (2.2%)	0 (0.0%)	0.503
Absent	89 (97.8%)	73 (100.0%)	
**FGFR4 Mutation**			
Present	1 (1.1%)	0 (0.0%)	1
Absent	90 (98.9%)	73 (100.0%)	
**FLNA Mutation**			
Present	6 (6.6%)	2 (2.7%)	0.3012
Absent	85 (93.4%)	71 (97.3%)	
**FLNB Mutation**			
Present	0 (0.0%)	0 (0.0%)	1
Absent	91 (100.0%)	73 (100.0%)	
**FLNC Mutation**			
Present	5 (5.5%)	4 (5.5%)	1
Absent	86 (94.5%)	69 (94.5%)	
**HRAS Mutation**			
Present	1 (1.1%)	0 (0.0%)	1
Absent	90 (98.9%)	73 (100.0%)	
**JUND Mutation**			
Present	1 (1.1%)	0 (0.0%)	1
Absent	90 (98.9%)	73 (100.0%)	
**KRAS Mutation**			

Present	60 (65.9%)	46 (63.0%)	0.8224
Absent	31 (34.1%)	27 (37.0%)	
**MAP2K4 Mutation**			
Present	3 (3.3%)	1 (1.4%)	0.6296
Absent	88 (96.7%)	72 (98.6%)	
**MAP2K6 Mutation**			
Present	3 (3.3%)	0 (0.0%)	0.2545
Absent	88 (96.7%)	73 (100.0%)	
**MAP2K7 Mutation**			
Present	2 (2.2%)	1 (1.4%)	1
Absent	89 (97.8%)	72 (98.6%)	
**MAP3K1 Mutation**			
Present	3 (3.3%)	0 (0.0%)	0.2545
Absent	88 (96.7%)	73 (100.0%)	
**MAP3K10 Mutation**			
Present	2 (2.2%)	0 (0.0%)	0.503
Absent	89 (97.8%)	73 (100.0%)	
**MAP3K12 Mutation**			
Present	1 (1.1%)	0 (0.0%)	1
Absent	90 (98.9%)	73 (100.0%)	
**MAP3K13 Mutation**			
Present	1 (1.1%)	0 (0.0%)	1
Absent	90 (98.9%)	73 (100.0%)	
**MAP3K2 Mutation**			
Present	2 (2.2%)	0 (0.0%)	0.503
Absent	89 (97.8%)	73 (100.0%)	
**MAP3K4 Mutation**			
Present	1 (1.1%)	0 (0.0%)	1
Absent	90 (98.9%)	73 (100.0%)	
**MAP3K5 Mutation**			
Present	2 (2.2%)	0 (0.0%)	0.503
Absent	89 (97.8%)	73 (100.0%)	
**MAP3K6 Mutation**			
Present	1 (1.1%)	0 (0.0%)	1
Absent	90 (98.9%)	73 (100.0%)	
**MAP4K1 Mutation**			
Present	2 (2.2%)	1 (1.4%)	1
Absent	89 (97.8%)	72 (98.6%)	
**MAP4K2 Mutation**			
Present	4 (4.4%)	0 (0.0%)	0.1295

Absent	87 (95.6%)	73 (100.0%)	
MAP4K3 Mutation			
Present	2 (2.2%)	0 (0.0%)	0.503
Absent	89 (97.8%)	73 (100.0%)	
MAP4K4 Mutation			
Present	1 (1.1%)	0 (0.0%)	1
Absent	90 (98.9%)	73 (100.0%)	
MAPK10 Mutation			
Present	0 (0.0%)	1 (1.4%)	0.4451
Absent	91 (100.0%)	72 (98.6%)	
MAPK11 Mutation			
Present	1 (1.1%)	0 (0.0%)	1
Absent	90 (98.9%)	73 (100.0%)	
MAPK12 Mutation			
Present	1 (1.1%)	0 (0.0%)	1
Absent	90 (98.9%)	73 (100.0%)	
MAPK13 Mutation			
Present	0 (0.0%)	1 (1.4%)	0.4451
Absent	91 (100.0%)	72 (98.6%)	
MOS Mutation			
Present	1 (1.1%)	0 (0.0%)	1
Absent	90 (98.9%)	73 (100.0%)	
MYC Mutation			
Present	0 (0.0%)	0 (0.0%)	1
Absent	91 (100.0%)	73 (100.0%)	
NF1 Mutation			
Present	2 (2.2%)	2 (2.7%)	1
Absent	89 (97.8%)	71 (97.3%)	
NFATC2 Mutation			
Present	3 (3.3%)	0 (0.0%)	0.2545
Absent	88 (96.7%)	73 (100.0%)	
NRAS Mutation			
Present	0 (0.0%)	0 (0.0%)	1
Absent	91 (100.0%)	73 (100.0%)	
NTRK1 Mutation			
Present	1 (1.1%)	1 (1.4%)	1
Absent	90 (98.9%)	72 (98.6%)	
NTRK2 Mutation			
Present	2 (2.2%)	0 (0.0%)	0.503
Absent	89 (97.8%)	73 (100.0%)	

**PAK2 Mutation**			
Present	2 (2.2%)	0 (0.0%)	0.503
Absent	89 (97.8%)	73 (100.0%)	
**PDGFRA Mutation**			
Present	2 (2.2%)	0 (0.0%)	0.503
Absent	89 (97.8%)	73 (100.0%)	
**PDGFRB Mutation**			
Present	1 (1.1%)	1 (1.4%)	1
Absent	90 (98.9%)	72 (98.6%)	
**PLA2G6 Mutation**			
Present	3 (3.3%)	0 (0.0%)	0.2545
Absent	88 (96.7%)	73 (100.0%)	
**PRKACB Mutation**			
Present	0 (0.0%)	0 (0.0%)	1
Absent	91 (100.0%)	73 (100.0%)	
**RAC2 Mutation**			
Present	1 (1.1%)	0 (0.0%)	1
Absent	90 (98.9%)	73 (100.0%)	
**RAPGEF2 Mutation**			
Present	2 (2.2%)	2 (2.7%)	1
Absent	89 (97.8%)	71 (97.3%)	
**RASA1 Mutation**			
Present	1 (1.1%)	1 (1.4%)	1
Absent	90 (98.9%)	72 (98.6%)	
**RPS6KA4 Mutation**			
Present	3 (3.3%)	0 (0.0%)	0.2545
Absent	88 (96.7%)	73 (100.0%)	
**RPS6KA6 Mutation**			
Present	2 (2.2%)	1 (1.4%)	1
Absent	89 (97.8%)	72 (98.6%)	
**SOS1 Mutation**			
Present	2 (2.2%)	0 (0.0%)	0.503
Absent	89 (97.8%)	73 (100.0%)	
**SOS2 Mutation**			
Present	1 (1.1%)	0 (0.0%)	1
Absent	90 (98.9%)	73 (100.0%)	
**TGFB1 Mutation**			
Present	1 (1.1%)	0 (0.0%)	1
Absent	90 (98.9%)	73 (100.0%)	
**TGFBR1 Mutation**			

Present	4 (4.4%)	0 (0.0%)	0.1295
Absent	87 (95.6%)	73 (100.0%)	
**TGFBR2 Mutation**			
Present	8 (8.8%)	1 (1.4%)	**0.0439**
Absent	83 (91.2%)	72 (98.6%)	
**TP53 Mutation**			
Present	52 (57.1%)	44 (60.3%)	0.8064
Absent	39 (42.9%)	29 (39.7%)	
**TRAF2 Mutation**			
Present	1 (1.1%)	0 (0.0%)	1
Absent	90 (98.9%)	73 (100.0%)	

Table S7. Comparison of Early-Onset PDAC Patients Versus Late-Onset PDAC Patients Treated with Gemcitabine.

MAPK Pathway			
**Gene**	**Early-Onset Treated with Gemcitabine n (%)**	**Late-Onset Treated with Gemcitabine n (%)**	**p-value**
**ACVR1 Mutation**			
Present	0 (0.0%)	0 (0.0%)	1
Absent	15 (100.0%)	91 (100.0%)	
**AKT1 Mutation**			
Present	0 (0.0%)	0 (0.0%)	1
Absent	15 (100.0%)	91 (100.0%)	
**AKT2 Mutation**			
Present	0 (0.0%)	1 (1.1%)	1
Absent	15 (100.0%)	90 (98.9%)	
**AKT3 Mutation**			
Present	0 (0.0%)	1 (1.1%)	1
Absent	15 (100.0%)	90 (98.9%)	
**BRAF Mutation**			
Present	0 (0.0%)	0 (0.0%)	1
Absent	15 (100.0%)	91 (100.0%)	
**CACNA1A Mutation**			
Present	1 (6.7%)	5 (5.5%)	1
Absent	14 (93.3%)	86 (94.5%)	
**CACNA1B Mutation**			
Present	0 (0.0%)	7 (7.7%)	0.5897
Absent	15 (100.0%)	84 (92.3%)	
**CACNA1C Mutation**			
Present	0 (0.0%)	9 (9.9%)	0.353

Absent	15 (100.0%)	82 (90.1%)	
CACNA1D Mutation			
Present	1 (6.7%)	5 (5.5%)	1
Absent	14 (93.3%)	86 (94.5%)	
CACNA1E Mutation			
Present	0 (0.0%)	7 (7.7%)	0.5897
Absent	15 (100.0%)	84 (92.3%)	
CACNA1F Mutation			
Present	0 (0.0%)	1 (1.1%)	1
Absent	15 (100.0%)	90 (98.9%)	
CACNA1G Mutation			
Present	1 (6.7%)	5 (5.5%)	1
Absent	14 (93.3%)	86 (94.5%)	
CACNA1H Mutation			
Present	0 (0.0%)	3 (3.3%)	1
Absent	15 (100.0%)	88 (96.7%)	
CACNA1I Mutation			
Present	0 (0.0%)	4 (4.4%)	1
Absent	15 (100.0%)	87 (95.6%)	
CACNA1S Mutation			
Present	0 (0.0%)	5 (5.5%)	1
Absent	15 (100.0%)	86 (94.5%)	
CACNA2D1 Mutation			
Present	0 (0.0%)	0 (0.0%)	1
Absent	15 (100.0%)	91 (100.0%)	
CACNA2D3 Mutation			
Present	0 (0.0%)	0 (0.0%)	1
Absent	15 (100.0%)	91 (100.0%)	
CACNA2D4 Mutation			
Present	0 (0.0%)	0 (0.0%)	1
Absent	15 (100.0%)	91 (100.0%)	
CACNB1 Mutation			
Present	0 (0.0%)	0 (0.0%)	1
Absent	15 (100.0%)	91 (100.0%)	
CACNB2 Mutation			
Present	0 (0.0%)	0 (0.0%)	1
Absent	15 (100.0%)	91 (100.0%)	
CACNG1 Mutation			
Present	0 (0.0%)	1 (1.1%)	1
Absent	15 (100.0%)	90 (98.9%)	

CACNG2 Mutation			
Present	0 (0.0%)	2 (2.2%)	1
Absent	15 (100.0%)	89 (97.8%)	
CACNG3 Mutation			
Present	1 (6.7%)	0 (0.0%)	0.1415
Absent	14 (93.3%)	91 (100.0%)	
CACNB2 Mutation			
Present	0 (0.0%)	1 (1.1%)	1
Absent	15 (100.0%)	90 (98.9%)	
CDC42 Mutation			
Present	0 (0.0%)	0 (0.0%)	1
Absent	15 (100.0%)	91 (100.0%)	
CRK Mutation			
Present	0 (0.0%)	0 (0.0%)	1
Absent	15 (100.0%)	91 (100.0%)	
DAXX Mutation			
Present	0 (0.0%)	2 (2.2%)	1
Absent	15 (100.0%)	89 (97.8%)	
DUSP6 Mutation			
Present	0 (0.0%)	2 (2.2%)	1
Absent	15 (100.0%)	89 (97.8%)	
DUSP7 Mutation			
Present	0 (0.0%)	2 (2.2%)	1
Absent	15 (100.0%)	89 (97.8%)	
DUSP10 Mutation			
Present	0 (0.0%)	1 (1.1%)	1
Absent	15 (100.0%)	90 (98.9%)	
DUSP14 Mutation			
Present	0 (0.0%)	1 (1.1%)	1
Absent	15 (100.0%)	90 (98.9%)	
EGFR Mutation			
Present	0 (0.0%)	2 (2.2%)	1
Absent	15 (100.0%)	89 (97.8%)	
FGF12 Mutation			
Present	0 (0.0%)	1 (1.1%)	1
Absent	15 (100.0%)	90 (98.9%)	
FGF13 Mutation			
Present	0 (0.0%)	1 (1.1%)	1
Absent	15 (100.0%)	90 (98.9%)	
FGF14 Mutation			

Present	0 (0.0%)	2 (2.2%)	1
Absent	15 (100.0%)	89 (97.8%)	
**FGF17 Mutation**			
Present	0 (0.0%)	1 (1.1%)	1
Absent	15 (100.0%)	90 (98.9%)	
**FGF2 Mutation**			
Present	0 (0.0%)	1 (1.1%)	1
Absent	15 (100.0%)	90 (98.9%)	
**FGF23 Mutation**			
Present	0 (0.0%)	1 (1.1%)	1
Absent	15 (100.0%)	90 (98.9%)	
**FGF6 Mutation**			
Present	0 (0.0%)	1 (1.1%)	1
Absent	15 (100.0%)	90 (98.9%)	
**FGFR3 Mutation**			
Present	0 (0.0%)	2 (2.2%)	1
Absent	15 (100.0%)	89 (97.8%)	
**FGFR4 Mutation**			
Present	0 (0.0%)	1 (1.1%)	1
Absent	15 (100.0%)	90 (98.9%)	
**FLNA Mutation**			
Present	2 (13.3%)	6 (6.6%)	0.3156
Absent	13 (86.7%)	85 (93.4%)	
**FLNB Mutation**			
Present	2 (13.3%)	0 (0.0%)	**0.0189**
Absent	13 (86.7%)	91 (100.0%)	
**FLNC Mutation**			
Present	1 (6.7%)	5 (5.5%)	1
Absent	14 (93.3%)	86 (94.5%)	
**HRAS Mutation**			
Present	0 (0.0%)	1 (1.1%)	1
Absent	15 (100.0%)	90 (98.9%)	
**JUND Mutation**			
Present	0 (0.0%)	1 (1.1%)	1
Absent	15 (100.0%)	90 (98.9%)	
**KRAS Mutation**			
Present	11 (73.3%)	60 (65.9%)	0.7688
Absent	4 (26.7%)	31 (34.1%)	
**MAP2K4 Mutation**			
Present	0 (0.0%)	3 (3.3%)	1

Absent	15 (100.0%)	88 (96.7%)	
**MAP2K6 Mutation**			
Present	0 (0.0%)	3 (3.3%)	1
Absent	15 (100.0%)	88 (96.7%)	
**MAP2K7 Mutation**			
Present	0 (0.0%)	2 (2.2%)	1
Absent	15 (100.0%)	89 (97.8%)	
**MAP3K1 Mutation**			
Present	0 (0.0%)	3 (3.3%)	1
Absent	15 (100.0%)	88 (96.7%)	
**MAP3K10 Mutation**			
Present	0 (0.0%)	2 (2.2%)	1
Absent	15 (100.0%)	89 (97.8%)	
**MAP3K12 Mutation**			
Present	0 (0.0%)	1 (1.1%)	1
Absent	15 (100.0%)	90 (98.9%)	
**MAP3K13 Mutation**			
Present	0 (0.0%)	1 (1.1%)	1
Absent	15 (100.0%)	90 (98.9%)	
**MAP3K2 Mutation**			
Present	0 (0.0%)	2 (2.2%)	1
Absent	15 (100.0%)	89 (97.8%)	
**MAP3K4 Mutation**			
Present	0 (0.0%)	1 (1.1%)	1
Absent	15 (100.0%)	90 (98.9%)	
**MAP3K5 Mutation**			
Present	0 (0.0%)	2 (2.2%)	1
Absent	15 (100.0%)	89 (97.8%)	
**MAP3K6 Mutation**			
Present	0 (0.0%)	1 (1.1%)	1
Absent	15 (100.0%)	90 (98.9%)	
**MAP4K1 Mutation**			
Present	0 (0.0%)	2 (2.2%)	1
Absent	15 (100.0%)	89 (97.8%)	
**MAP4K2 Mutation**			
Present	0 (0.0%)	4 (4.4%)	1
Absent	15 (100.0%)	87 (95.6%)	
**MAP4K3 Mutation**			
Present	0 (0.0%)	2 (2.2%)	1
Absent	15 (100.0%)	89 (97.8%)	

**MAP4K4 Mutation**			
Present	0 (0.0%)	1 (1.1%)	1
Absent	15 (100.0%)	90 (98.9%)	
**MAPK10 Mutation**			
Present	0 (0.0%)	0 (0.0%)	1
Absent	15 (100.0%)	91 (100.0%)	
**MAPK11 Mutation**			
Present	0 (0.0%)	1 (1.1%)	1
Absent	15 (100.0%)	90 (98.9%)	
**MAPK12 Mutation**			
Present	0 (0.0%)	1 (1.1%)	1
Absent	15 (100.0%)	90 (98.9%)	
**MAPK13 Mutation**			
Present	0 (0.0%)	0 (0.0%)	1
Absent	15 (100.0%)	91 (100.0%)	
**MOS Mutation**			
Present	1 (6.7%)	1 (1.1%)	0.2642
Absent	14 (93.3%)	90 (98.9%)	
**MYC Mutation**			
Present	0 (0.0%)	0 (0.0%)	1
Absent	15 (100.0%)	91 (100.0%)	
**NF1 Mutation**			
Present	0 (0.0%)	2 (2.2%)	1
Absent	15 (100.0%)	89 (97.8%)	
**NFATC2 Mutation**			
Present	1 (6.7%)	3 (3.3%)	0.462
Absent	14 (93.3%)	88 (96.7%)	
**NRAS Mutation**			
Present	0 (0.0%)	0 (0.0%)	1
Absent	15 (100.0%)	91 (100.0%)	
**NTRK1 Mutation**			
Present	1 (6.7%)	1 (1.1%)	0.0829
Absent	3 (20.0%)	90 (98.9%)	
**NTRK2 Mutation**			
Present	0 (0.0%)	2 (2.2%)	1
Absent	15 (100.0%)	89 (97.8%)	
**PAK2 Mutation**			
Present	0 (0.0%)	2 (2.2%)	1
Absent	15 (100.0%)	89 (97.8%)	
**PDGFRA Mutation**			

Present	0 (0.0%)	2 (2.2%)	1
Absent	15 (100.0%)	89 (97.8%)	
**PDGFRB Mutation**			
Present	0 (0.0%)	1 (1.1%)	1
Absent	15 (100.0%)	90 (98.9%)	
**PLA2G6 Mutation**			
Present	0 (0.0%)	3 (3.3%)	1
Absent	15 (100.0%)	88 (96.7%)	
**PRKACB Mutation**			
Present	1 (6.7%)	0 (0.0%)	0.1415
Absent	14 (93.3%)	91 (100.0%)	
**RAC2 Mutation**			
Present	0 (0.0%)	1 (1.1%)	1
Absent	15 (100.0%)	90 (98.9%)	
**RAPGEF2 Mutation**			
Present	0 (0.0%)	2 (2.2%)	1
Absent	15 (100.0%)	89 (97.8%)	
**RASA1 Mutation**			
Present	0 (0.0%)	1 (1.1%)	1
Absent	15 (100.0%)	90 (98.9%)	
**RPS6KA4 Mutation**			
Present	0 (0.0%)	3 (3.3%)	1
Absent	15 (100.0%)	88 (96.7%)	
**RPS6KA6 Mutation**			
Present	0 (0.0%)	2 (2.2%)	1
Absent	15 (100.0%)	89 (97.8%)	
**SOS1 Mutation**			
Present	0 (0.0%)	2 (2.2%)	1
Absent	15 (100.0%)	89 (97.8%)	
**SOS2 Mutation**			
Present	0 (0.0%)	1 (1.1%)	1
Absent	15 (100.0%)	90 (98.9%)	
**TGFB1 Mutation**			
Present	0 (0.0%)	1 (1.1%)	1
Absent	15 (100.0%)	90 (98.9%)	
**TGFBR1 Mutation**			
Present	0 (0.0%)	4 (4.4%)	1
Absent	15 (100.0%)	87 (95.6%)	
**TGFBR2 Mutation**			
Present	1 (6.7%)	8 (8.8%)	1

Absent	14 (93.3%)	83 (91.2%)	
**TP53 Mutation**			
Present	13 (86.7%)	52 (57.1%)	**0.0431**
Absent	2 (13.3%)	39 (42.9%)	
**TRAF2 Mutation**			
Present	0 (0.0%)	1 (1.1%)	1
Absent	15 (100.0%)	90 (98.9%)	

Table S8. Comparison of Early-Onset PDAC Patients Versus Late-Onset PDAC Patients Not Treated with Gemcitabine.

MAPK Pathway			
Gene	Early-Onset<br>Not Treated with Gemcitabine<br>n (%)	Late-Onset<br>Not Treated with Gemcitabine<br>n (%)	p-value
**ACVR1 Mutation**			
Present	0 (0.0%)	0 (0.0%)	1
Absent	5 (100.0%)	73 (100.0%)	
**AKT1 Mutation**			
Present	0 (0.0%)	0 (0.0%)	1
Absent	5 (100.0%)	73 (100.0%)	
**AKT2 Mutation**			
Present	0 (0.0%)	0 (0.0%)	1
Absent	5 (100.0%)	73 (100.0%)	
**AKT3 Mutation**			
Present	0 (0.0%)	0 (0.0%)	1
Absent	5 (100.0%)	73 (100.0%)	
**BRAF Mutation**			
Present	0 (0.0%)	2 (2.7%)	1
Absent	5 (100.0%)	71 (97.3%)	
**CACNA1A Mutation**			
Present	0 (0.0%)	1 (1.4%)	1
Absent	5 (100.0%)	72 (98.6%)	
**CACNA1B Mutation**			
Present	0 (0.0%)	4 (5.5%)	1
Absent	5 (100.0%)	69 (94.5%)	
**CACNA1C Mutation**			
Present	0 (0.0%)	0 (0.0%)	1
Absent	5 (100.0%)	73 (100.0%)	
**CACNA1D Mutation**			
Present	0 (0.0%)	1 (1.4%)	1
Absent	5 (100.0%)	72 (98.6%)	

**CACNA1E Mutation**			
Present	0 (0.0%)	0 (0.0%)	1
Absent	5 (100.0%)	73 (100.0%)	
**CACNA1F Mutation**			
Present	0 (0.0%)	1 (1.4%)	1
Absent	5 (100.0%)	72 (98.6%)	
**CACNA1G Mutation**			
Present	0 (0.0%)	1 (1.4%)	1
Absent	5 (100.0%)	72 (98.6%)	
**CACNA1H Mutation**			
Present	0 (0.0%)	1 (1.4%)	1
Absent	5 (100.0%)	72 (98.6%)	
**CACNA1I Mutation**			
Present	0 (0.0%)	2 (2.7%)	1
Absent	5 (100.0%)	71 (97.3%)	
**CACNA1S Mutation**			
Present	0 (0.0%)	1 (1.4%)	1
Absent	5 (100.0%)	72 (98.6%)	
**CACNA2D1 Mutation**			
Present	2 (40.0%)	1 (1.4%)	**0.0097**
Absent	3 (60.0%)	72 (98.6%)	
**CACNA2D3 Mutation**			
Present	2 (40.0%)	1 (1.4%)	**0.0097**
Absent	3 (60.0%)	72 (98.6%)	
**CACNA2D4 Mutation**			
Present	2 (40.0%)	1 (1.4%)	**0.0097**
Absent	3 (60.0%)	72 (98.6%)	
**CACNB1 Mutation**			
Present	0 (0.0%)	2 (2.7%)	1
Absent	5 (100.0%)	71 (97.3%)	
**CACNB2 Mutation**			
Present	0 (0.0%)	2 (2.7%)	1
Absent	5 (100.0%)	71 (97.3%)	
**CACNG1 Mutation**			
Present	0 (0.0%)	0 (0.0%)	1
Absent	5 (100.0%)	73 (100.0%)	
**CACNG2 Mutation**			
Present	0 (0.0%)	0 (0.0%)	1
Absent	5 (100.0%)	73 (100.0%)	
**CACNG3 Mutation**			

Present	0 (0.0%)	1 (1.4%)	1
Absent	5 (100.0%)	72 (98.6%)	
**CACNB2 Mutation**			
Present	0 (0.0%)	0 (0.0%)	1
Absent	5 (100.0%)	73 (100.0%)	
**CDC42 Mutation**			
Present	0 (0.0%)	1 (1.4%)	1
Absent	5 (100.0%)	72 (98.6%)	
**CRK Mutation**			
Present	0 (0.0%)	0 (0.0%)	1
Absent	5 (100.0%)	73 (100.0%)	
**DAXX Mutation**			
Present	0 (0.0%)	1 (1.4%)	1
Absent	5 (100.0%)	72 (98.6%)	
**DUSP6 Mutation**			
Present	0 (0.0%)	0 (0.0%)	1
Absent	5 (100.0%)	73 (100.0%)	
**DUSP7 Mutation**			
Present	0 (0.0%)	0 (0.0%)	1
Absent	5 (100.0%)	73 (100.0%)	
**DUSP10 Mutation**			
Present	0 (0.0%)	0 (0.0%)	1
Absent	5 (100.0%)	73 (100.0%)	
**DUSP14 Mutation**			
Present	0 (0.0%)	0 (0.0%)	1
Absent	5 (100.0%)	73 (100.0%)	
**EGFR Mutation**			
Present	0 (0.0%)	0 (0.0%)	1
Absent	5 (100.0%)	73 (100.0%)	
**FGF12 Mutation**			
Present	0 (0.0%)	0 (0.0%)	1
Absent	5 (100.0%)	73 (100.0%)	
**FGF13 Mutation**			
Present	0 (0.0%)	1 (1.4%)	1
Absent	5 (100.0%)	72 (98.6%)	
**FGF14 Mutation**			
Present	0 (0.0%)	0 (0.0%)	1
Absent	5 (100.0%)	73 (100.0%)	
**FGF17 Mutation**			
Present	0 (0.0%)	0 (0.0%)	1

Absent	5 (100.0%)	73 (100.0%)	
**FGF2 Mutation**			
Present	0 (0.0%)	0 (0.0%)	1
Absent	5 (100.0%)	73 (100.0%)	
**FGF23 Mutation**			
Present	0 (0.0%)	0 (0.0%)	1
Absent	5 (100.0%)	73 (100.0%)	
**FGF6 Mutation**			
Present	1 (20.0%)	0 (0.0%)	0.0641
Absent	4 (80.0%)	73 (100.0%)	
**FGFR3 Mutation**			
Present	0 (0.0%)	0 (0.0%)	1
Absent	5 (100.0%)	73 (100.0%)	
**FGFR4 Mutation**			
Present	0 (0.0%)	0 (0.0%)	1
Absent	5 (100.0%)	73 (100.0%)	
**FLNA Mutation**			
Present	0 (0.0%)	2 (2.7%)	1
Absent	5 (100.0%)	71 (97.3%)	
**FLNB Mutation**			
Present	0 (0.0%)	0 (0.0%)	1
Absent	5 (100.0%)	73 (100.0%)	
**FLNC Mutation**			
Present	0 (0.0%)	4 (5.5%)	1
Absent	5 (100.0%)	69 (94.5%)	
**HRAS Mutation**			
Present	0 (0.0%)	0 (0.0%)	1
Absent	5 (100.0%)	73 (100.0%)	
**JUND Mutation**			
Present	0 (0.0%)	0 (0.0%)	1
Absent	5 (100.0%)	73 (100.0%)	
**KRAS Mutation**			
Present	4 (80.0%)	46 (63.0%)	0.6491
Absent	1 (20.0%)	27 (37.0%)	
**MAP2K4 Mutation**			
Present	0 (0.0%)	1 (1.4%)	1
Absent	5 (100.0%)	72 (98.6%)	
**MAP2K6 Mutation**			
Present	0 (0.0%)	0 (0.0%)	1
Absent	5 (100.0%)	73 (100.0%)	

**MAP2K7 Mutation**			
Present	0 (0.0%)	1 (1.4%)	1
Absent	5 (100.0%)	72 (98.6%)	
**MAP3K1 Mutation**			
Present	0 (0.0%)	0 (0.0%)	1
Absent	5 (100.0%)	73 (100.0%)	
**MAP3K10 Mutation**			
Present	0 (0.0%)	0 (0.0%)	1
Absent	5 (100.0%)	73 (100.0%)	
**MAP3K12 Mutation**			
Present	0 (0.0%)	0 (0.0%)	1
Absent	5 (100.0%)	73 (100.0%)	
**MAP3K13 Mutation**			
Present	0 (0.0%)	0 (0.0%)	1
Absent	5 (100.0%)	73 (100.0%)	
**MAP3K2 Mutation**			
Present	0 (0.0%)	0 (0.0%)	1
Absent	5 (100.0%)	73 (100.0%)	
**MAP3K4 Mutation**			
Present	0 (0.0%)	0 (0.0%)	1
Absent	5 (100.0%)	73 (100.0%)	
**MAP3K5 Mutation**			
Present	0 (0.0%)	0 (0.0%)	1
Absent	5 (100.0%)	73 (100.0%)	
**MAP3K6 Mutation**			
Present	0 (0.0%)	0 (0.0%)	1
Absent	5 (100.0%)	73 (100.0%)	
**MAP4K1 Mutation**			
Present	0 (0.0%)	1 (1.4%)	1
Absent	5 (100.0%)	72 (98.6%)	
**MAP4K2 Mutation**			
Present	0 (0.0%)	0 (0.0%)	1
Absent	5 (100.0%)	73 (100.0%)	
**MAP4K3 Mutation**			
Present	0 (0.0%)	0 (0.0%)	1
Absent	5 (100.0%)	73 (100.0%)	
**MAP4K4 Mutation**			
Present	0 (0.0%)	0 (0.0%)	1
Absent	5 (100.0%)	73 (100.0%)	
**MAPK10 Mutation**			

Present	0 (0.0%)	1 (1.4%)	1
Absent	5 (100.0%)	72 (98.6%)	
**MAPK11 Mutation**			
Present	0 (0.0%)	0 (0.0%)	1
Absent	5 (100.0%)	73 (100.0%)	
**MAPK12 Mutation**			
Present	0 (0.0%)	0 (0.0%)	1
Absent	5 (100.0%)	73 (100.0%)	
**MAPK13 Mutation**			
Present	0 (0.0%)	1 (1.4%)	1
Absent	5 (100.0%)	72 (98.6%)	
**MOS Mutation**			
Present	0 (0.0%)	0 (0.0%)	1
Absent	5 (100.0%)	73 (100.0%)	
**MYC Mutation**			
Present	0 (0.0%)	0 (0.0%)	1
Absent	5 (100.0%)	73 (100.0%)	
**NF1 Mutation**			
Present	0 (0.0%)	2 (2.7%)	1
Absent	5 (100.0%)	71 (97.3%)	
**NFATC2 Mutation**			
Present	0 (0.0%)	0 (0.0%)	1
Absent	5 (100.0%)	73 (100.0%)	
**NRAS Mutation**			
Present	0 (0.0%)	0 (0.0%)	1
Absent	5 (100.0%)	73 (100.0%)	
**NTRK1 Mutation**			
Present	0 (0.0%)	1 (1.4%)	1
Absent	5 (100.0%)	72 (98.6%)	
**NTRK2 Mutation**			
Present	0 (0.0%)	0 (0.0%)	1
Absent	5 (100.0%)	73 (100.0%)	
**PAK2 Mutation**			
Present	0 (0.0%)	0 (0.0%)	1
Absent	5 (100.0%)	73 (100.0%)	
**PDGFRA Mutation**			
Present	0 (0.0%)	0 (0.0%)	1
Absent	5 (100.0%)	73 (100.0%)	
**PDGFRB Mutation**			
Present	0 (0.0%)	1 (1.4%)	1

Absent	5 (100.0%)	72 (98.6%)	
**PLA2G6 Mutation**			
Present	0 (0.0%)	0 (0.0%)	1
Absent	5 (100.0%)	73 (100.0%)	
**PRKACB Mutation**			
Present	0 (0.0%)	0 (0.0%)	1
Absent	5 (100.0%)	73 (100.0%)	
**RAC2 Mutation**			
Present	0 (0.0%)	0 (0.0%)	1
Absent	5 (100.0%)	73 (100.0%)	
**RAPGEF2 Mutation**			
Present	0 (0.0%)	2 (2.7%)	1
Absent	5 (100.0%)	71 (97.3%)	
**RASA1 Mutation**			
Present	0 (0.0%)	1 (1.4%)	1
Absent	5 (100.0%)	72 (98.6%)	
**RPS6KA4 Mutation**			
Present	0 (0.0%)	0 (0.0%)	1
Absent	5 (100.0%)	73 (100.0%)	
**RPS6KA6 Mutation**			
Present	0 (0.0%)	1 (1.4%)	1
Absent	5 (100.0%)	72 (98.6%)	
**SOS1 Mutation**			
Present	0 (0.0%)	0 (0.0%)	1
Absent	5 (100.0%)	73 (100.0%)	
**SOS2 Mutation**			
Present	0 (0.0%)	0 (0.0%)	1
Absent	5 (100.0%)	73 (100.0%)	
**TGFB1 Mutation**			
Present	0 (0.0%)	0 (0.0%)	1
Absent	5 (100.0%)	73 (100.0%)	
**TGFBR1 Mutation**			
Present	0 (0.0%)	0 (0.0%)	1
Absent	5 (100.0%)	73 (100.0%)	
**TGFBR2 Mutation**			
Present	0 (0.0%)	1 (1.4%)	1
Absent	5 (100.0%)	72 (98.6%)	
**TP53 Mutation**			
Present	2 (40.0%)	44 (60.3%)	0.396
Absent	3 (60.0%)	29 (39.7%)	

TRAF2 Mutation			
Present	0 (0.0%)	0 (0.0%)	1
Absent	5 (100.0%)	73 (100.0%)	

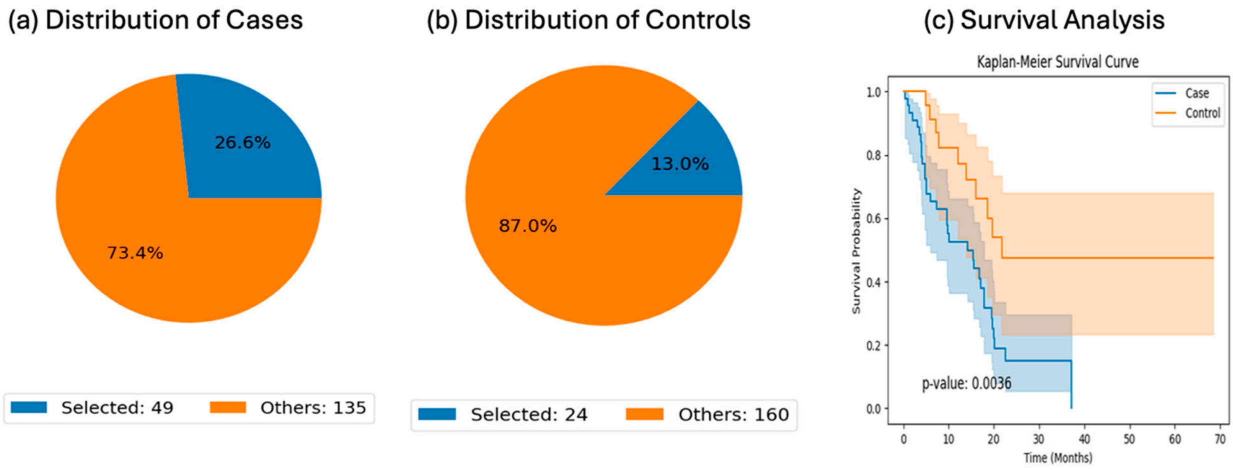

Figure S1. Conversational AI–enabled cohort definition and survival analysis in late-onset PDAC patients not treated with gemcitabine, stratified by RTK–RAS pathway status. This figure illustrates AI-guided cohort selection and outcome comparison within late-onset PDAC patients who did not receive gemcitabine. Using natural language criteria, the AI-HOPE-RTK-RAS agent identified (a) a case cohort comprising late-onset, non–gemcitabine-treated patients harboring RTK–RAS pathway alterations (n = 49; 26.6% of the dataset), and (b) a control cohort of similarly defined patients lacking RTK–RAS alterations (n = 24; 13.0%). Pie charts display the proportional representation of selected versus non-selected samples within the full cohort. (c) Kaplan–Meier overall survival analysis demonstrated a statistically significant difference between groups (log-rank p = 0.0036), with RTK–RAS–altered tumors associated with reduced survival compared with pathway–wild-type tumors. Shaded areas denote 95% confidence intervals. These results underscore the prognostic relevance of RTK–RAS pathway status in late-onset PDAC outside the context of gemcitabine exposure and highlight the capacity of conversational AI to reproducibly construct clinically meaningful cohorts.

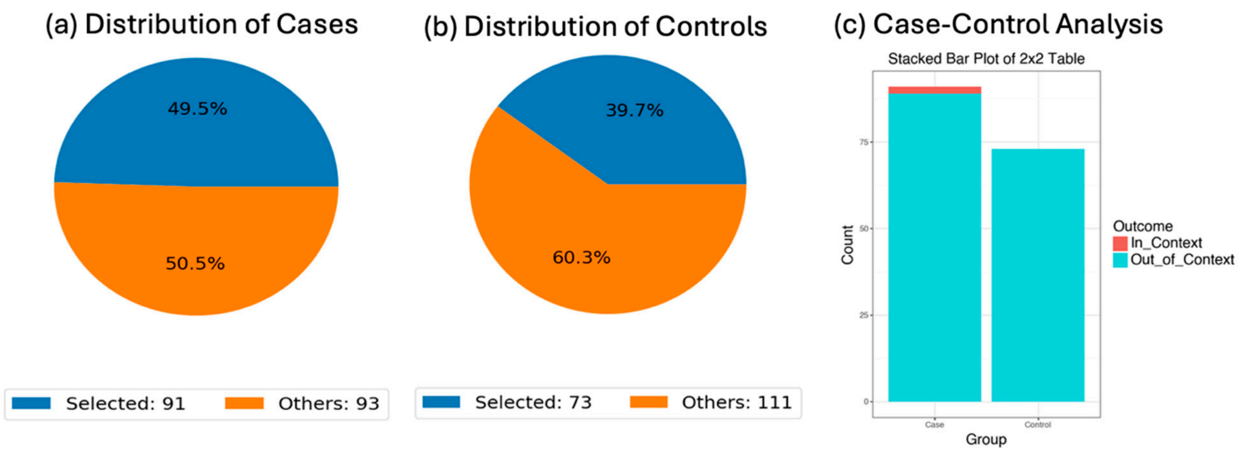

Figure S2. Conversational AI–guided comparison of ERBB2 mutation frequency in late-onset PDAC patients stratified by gemcitabine exposure. This figure illustrates an AI-enabled odds ratio analysis evaluating whether ERBB2 mutation prevalence differs between late-onset PDAC patients treated with gemcitabine (case cohort; n = 91) and those not treated with gemcitabine (control cohort; n = 73). Cohorts were constructed using structured natural language criteria within the AI-HOPE-RTK-RAS framework. Pie charts display the proportion of selected samples within each group relative to the total dataset. The stacked bar plot summarizes ERBB2-mutated ("In-Context") versus non-mutated ("Out-of-Context") samples across case and control cohorts. ERBB2 mutations were observed in 2.2% of gemcitabine-treated cases and 0.68% of non-treated controls. Fisher's exact test demonstrated no statistically significant difference between groups (p = 0.576), and the estimated odds ratio indicated no meaningful enrichment of ERBB2 mutations associated with gemcitabine exposure in late-onset PDAC. These findings suggest that ERBB2 mutation frequency does not substantially differ by gemcitabine treatment status within this age-defined subgroup and demonstrate the utility of conversational AI for rapid genomic frequency comparisons.

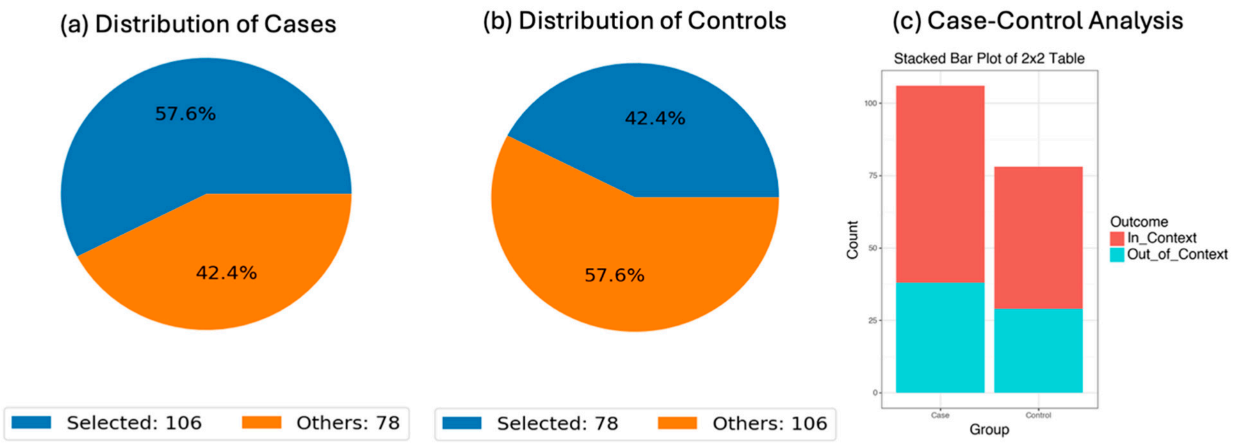

Figure S3. Conversational AI–enabled comparison of KRAS mutation prevalence by gemcitabine treatment status in PDAC. This figure depicts an AI-assisted odds ratio analysis evaluating whether KRAS mutation frequency differs between PDAC patients treated with gemcitabine (case cohort; n = 106) and those not treated with gemcitabine (control cohort; n = 78). Cohorts were defined using structured clinical criteria within the AI-HOPE-RTK-RAS framework. Pie charts illustrate the proportion of selected samples within each treatment group relative to the full dataset. The stacked bar plot summarizes KRAS-mutated ("In-Context") and KRAS–wild-type ("Out-of-Context") samples across treated and non-treated cohorts. KRAS mutations were highly prevalent in both groups (64.1% in treated vs. 62.8% in non-treated patients). Statistical testing demonstrated no significant difference in mutation frequency by gemcitabine exposure (Fisher's exact p = 0.976; odds ratio 1.059, 95% CI 0.577–1.943). These findings indicate that KRAS mutation prevalence is comparable regardless of gemcitabine treatment status, reinforcing its near-ubiquitous role in PDAC biology and highlighting the utility of conversational AI for rapid treatment-stratified genomic comparisons.

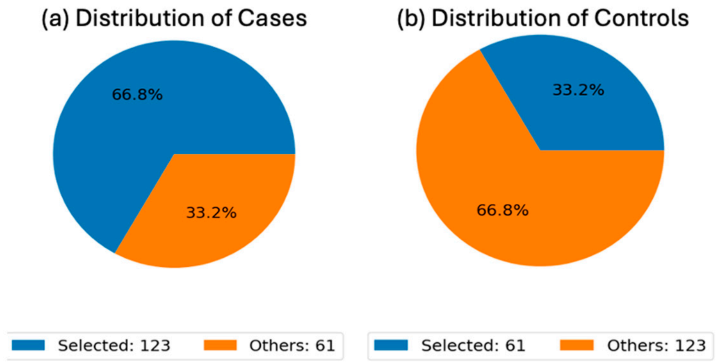

(c) Association Analysis

Attribute	Test	Statistic	P-value
RTKRAS_Pathway	Chi-square		6.18E-41
KRAS	Chi-square		1.22E-35
MAPK_Pathway	Chi-square		1.37E-20
Tumor Break Load	Mann-Whitney U	6135	3.63E-13
TP53_Pathway	Chi-square		1.67E-11
TP53	Chi-square		2.29E-10
TMB (nonsynonymous)	Mann-Whitney U	5469	2.98E-10
Winter Hypoxia Score	Mann-Whitney U	5490.5	1.66E-09
Mutation Count	Mann-Whitney U	4915.5	4.05E-09
Fraction Genome Altered	Mann-Whitney U	5624	8.74E-09
Buffa Hypoxia Score	Mann-Whitney U	5366	1.65E-08
Aneuploidy Score	Mann-Whitney U	4046.5	7.34E-07
Ragnum Hypoxia Score	Mann-Whitney U	5053	2.75E-06
In PanCan Pathway Analysis	Chi-square		6.80E-06
SMAD4	Chi-square		0.00013577
CDKN2A	Chi-square		0.00028093
Event	Chi-square		0.00037664
Overall Survival Status	Chi-square		0.00037664
TGF beta Pathway	Chi-square		0.00038961

Figure S4. Conversational AI-driven association analysis of clinical and molecular attributes linked to RTK-RAS pathway status in PDAC. This figure summarizes an AI-guided exploratory analysis comparing PDAC samples harboring RTK-RAS pathway alterations (case cohort; n = 123) with pathway-non-altered tumors (control cohort; n = 61). Panels (a) and (b) illustrate the proportional distribution of selected (in-context) and unselected samples within each cohort, demonstrating that RTK-RAS-altered tumors comprised 66.8% of the dataset, whereas 33.2% were pathway-non-altered. Panel (c) presents the results of a comprehensive association analysis integrating categorical (Chi-square) and continuous (Mann-Whitney U) tests to identify attributes significantly linked to RTK-RAS pathway status. As expected, strong associations were observed with KRAS mutation status and MAPK pathway alterations. Additional significant associations included TP53 mutation status, TP53 pathway involvement, SMAD4 and CDKN2A alterations, and TGFβ pathway status. Quantitative genomic features such as tumor mutation burden (nonsynonymous), total mutation count, fraction of genome altered, aneuploidy score, and multiple hypoxia signatures (Winter, Buffa, Ragnum) were also significantly enriched in RTK-RAS-altered tumors. Clinical outcome variables, including overall survival status and event occurrence, were likewise associated with pathway status. These AI-derived associations highlight a coordinated genomic, pathway-level, and microenvironmental signature linked to RTK-RAS alteration status in PDAC, demonstrating the capacity of conversational AI to rapidly uncover multidimensional clinical-molecular relationships.

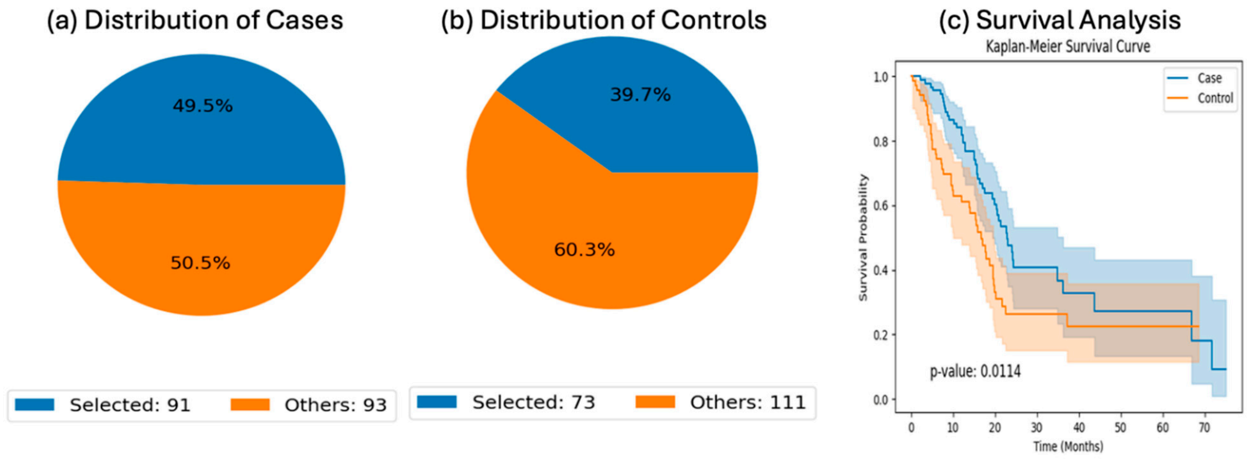

Figure S5. Conversational AI-driven cohort selection and survival analysis for late-onset PDAC stratified by MAPK pathway status and gemcitabine exposure. This figure demonstrates AI-enabled cohort construction and outcome comparison in late-onset PDAC. Using structured natural language queries, the AI-HOPE-MAPK module identified (a) a case cohort of gemcitabine-treated late-onset patients (n = 91; 49.5% of the dataset) and (b) a control cohort of non-gemcitabine-treated late-onset patients (n = 73; 39.7%). Pie charts depict the proportional distribution of selected versus non-selected samples within the full cohort. Panel (c) presents Kaplan-Meier overall survival curves comparing the two groups. A statistically significant difference in survival was observed (log-rank p = 0.0114), with gemcitabine-treated patients demonstrating distinct survival dynamics relative to untreated controls over time. Shaded regions indicate 95% confidence intervals. This figure highlights the capacity of conversational AI to rapidly define treatment-contextual cohorts and to generate reproducible survival analyses aligned with pathway-focused research questions in PDAC.

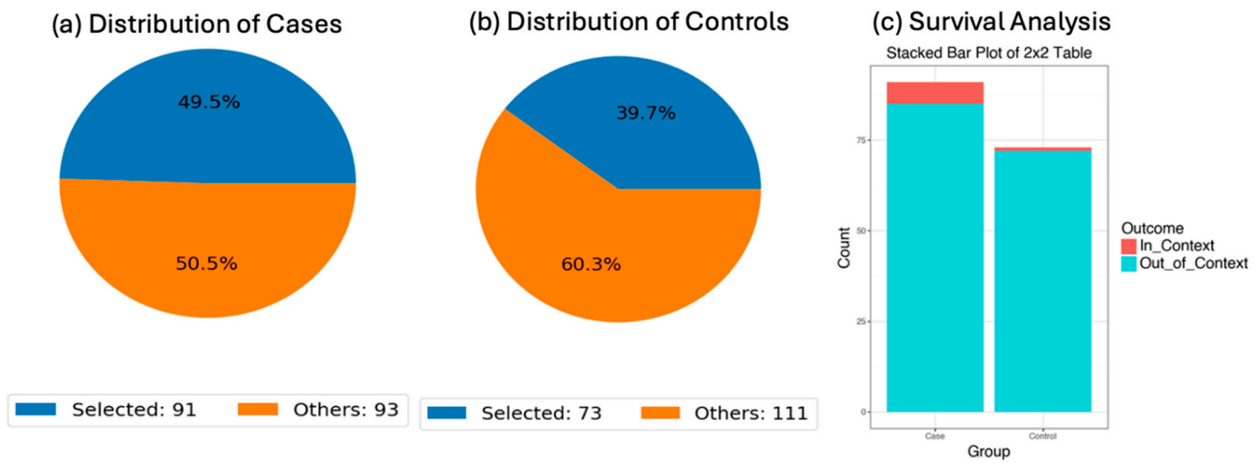

Figure S6. Conversational AI-based evaluation of TGFBR2 mutation frequency in late-onset PDAC stratified by gemcitabine exposure. This figure presents an AI-enabled odds ratio analysis assessing whether TGFBR2 mutation prevalence differs between late-onset PDAC patients treated with gemcitabine (case cohort; n = 91) and those not treated with gemcitabine (control cohort; n = 73). Cohorts were defined using structured clinical filters within the AI-HOPE-MAPK framework. Panels (a) and (b) display the distribution of selected ("in-context," TGFBR2-mutated) and unselected ("out-of-context," non-mutated) samples within each treatment group. The stacked bar plot summarizes mutation frequencies across case and control cohorts. TGFBR2 mutations were observed in 6.59% of gemcitabine-treated patients compared with 1.37% of non-treated patients. Statistical comparison using Fisher's exact test did not demonstrate a significant association between gemcitabine exposure and TGFBR2 mutation status ($p = 0.209$; odds ratio 5.082, 95% CI 0.598-43.204). These findings indicate that, although numerically higher in the treated cohort, TGFBR2 mutation frequency does not differ significantly by gemcitabine exposure in late-onset PDAC. The analysis highlights the ability of conversational AI to rapidly generate treatment-stratified genomic comparisons within pathway-focused investigations.

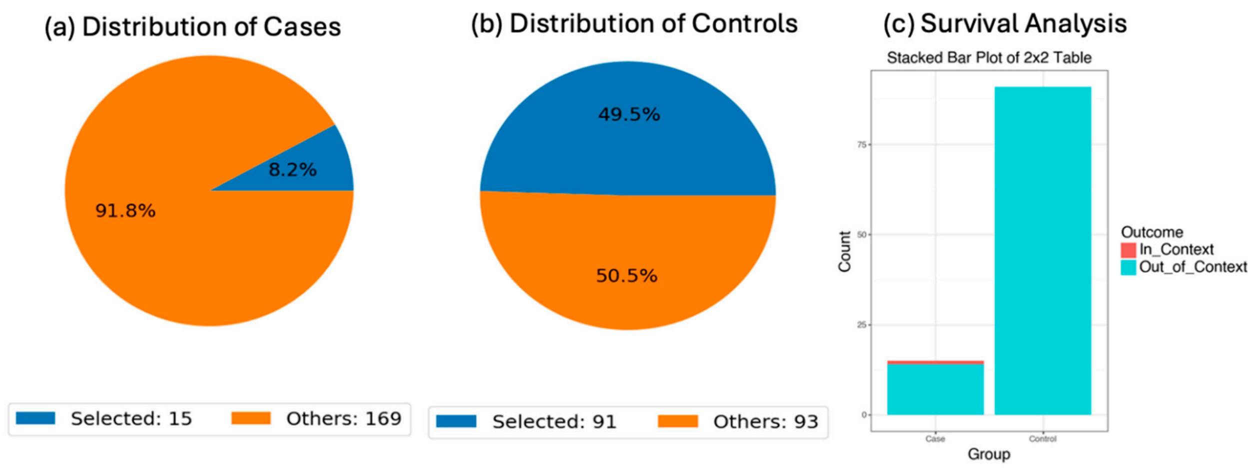

Figure S7. Conversational AI-enabled comparison of FLNB mutation frequency in gemcitabine-treated PDAC stratified by age at onset. This figure presents an AI-guided odds ratio analysis assessing whether FLNB mutation prevalence differs between early-onset (EO) and late-onset (LO) PDAC patients who received gemcitabine. The case cohort included EO gemcitabine-treated patients (n = 15), and the control cohort comprised LO gemcitabine-treated patients (n = 91), defined using structured clinical filters within the AI-HOPE-MAPK framework. Panels (a) and (b) illustrate the proportion of FLNB-mutated ("in-context") and non-mutated ("out-of-context") samples within each subgroup. FLNB mutations were observed in 6.67% of EO treated patients compared with 0.55% of LO treated patients. Panel (c) displays a stacked bar plot summarizing the 2×2 comparison. Statistical testing did not demonstrate a significant association between age group and FLNB mutation frequency in the gemcitabine-treated setting (Fisher's exact p = 0.301; odds ratio 13.0, 95% CI 0.416-405.866). Although numerically enriched in early-onset treated tumors, FLNB mutation prevalence did not reach statistical significance, likely reflecting small sample size. This analysis highlights the utility of conversational AI for rapidly performing age-stratified, treatment-specific genomic comparisons within MAPK pathway investigations.

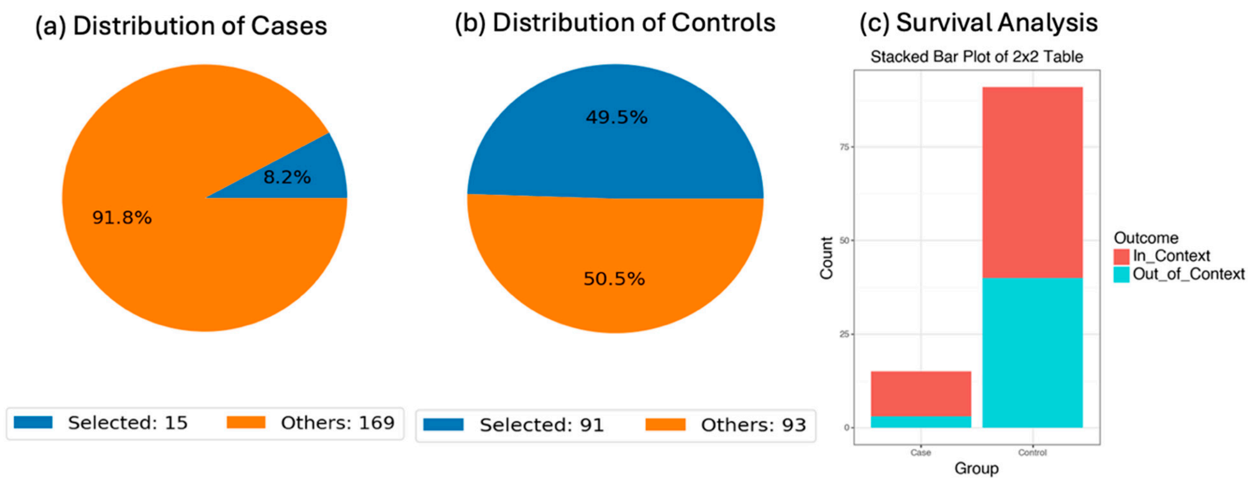

Figure S8. Conversational AI-driven evaluation of TP53 mutation frequency in gemcitabine-treated PDAC stratified by age at onset. This figure depicts an AI-enabled comparison of TP53 mutation prevalence between early-onset (EO) and late-onset (LO) PDAC patients who received gemcitabine. The case cohort included EO, gemcitabine-treated patients (n = 15), while the control cohort comprised LO, gemcitabine-treated patients (n = 91), identified through structured natural language queries within the AI-HOPE framework. Panels (a) and (b) present pie charts summarizing the proportion of TP53-mutated ("in-context") and non-mutated ("out-of-context") samples in each subgroup. TP53 alterations were observed in 80.0% of EO treated tumors compared with 56.0% of LO treated tumors. Panel (c) illustrates the corresponding 2×2 comparison using a stacked bar plot. Although TP53 mutations were numerically more frequent in early-onset treated patients, statistical testing did not demonstrate a significant association between age category and TP53 mutation status in the gemcitabine-treated setting (Chi-square p = 0.142; odds ratio 3.137, 95% CI 0.829-11.876). These findings suggest a potential enrichment of TP53 alterations in early-onset PDAC receiving gemcitabine; however, the lack of statistical significance and limited sample size warrant cautious interpretation. This example further highlights the capacity of conversational AI to rapidly perform age- and treatment-specific genomic comparisons within MAPK pathway-focused analyses.

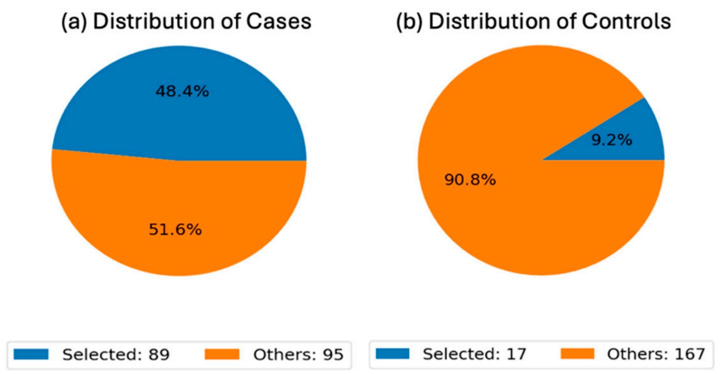

(c) Association Analysis

Attribute	Test	Statistic	P-value
MAPK_Pathway	Chi-square		2.93E-23
RTKRAS_Pathway	Chi-square		2.03E-09
TMB (nonsynonymous)	Mann-Whitney U	1229.5	5.41E-09
KRAS	Chi-square		9.28E-09
TP53_Pathway	Chi-square		1.09E-07
TP53	Chi-square		2.26E-07
Mutation Count	Mann-Whitney U	874.5	6.24E-07
In PanCan Pathway Analysis	Chi-square		1.03E-06
Tumor Break Load	Mann-Whitney U	1222.5	5.41E-06
Fraction Genome Altered	Mann-Whitney U	1209	9.45E-06
Aneuploidy Score	Mann-Whitney U	803	4.16E-05
Buffa Hypoxia Score	Mann-Whitney U	1168.5	6.20E-05
Winter Hypoxia Score	Mann-Whitney U	1158.5	9.11E-05
Ragnum Hypoxia Score	Mann-Whitney U	1110.5	0.00047941
TGFbeta_Pathway	Chi-square		0.00756946
SMAD4	Chi-square		0.04059825

Figure S9. AI-enabled identification of clinical and genomic features associated with MAPK pathway alterations in gemcitabine-treated PDAC. This figure summarizes a conversational AI-guided comparative analysis of PDAC tumors treated with gemcitabine, stratified by MAPK pathway status. The case cohort consisted of gemcitabine-treated tumors harboring MAPK pathway alterations (n = 89), while the control cohort included gemcitabine-treated, MAPK-non-altered tumors (n = 17). Panels (a) and (b) display the distribution of selected (MAPK-altered) and unselected samples within each subgroup, illustrating the predominance of pathway-altered tumors among gemcitabine-treated cases. Panel (c) presents the results of a comprehensive association analysis integrating Chi-square tests for categorical variables and Mann-Whitney U tests for continuous measures. Strong associations were observed between MAPK pathway alteration status and RTK-RAS pathway involvement, KRAS mutation status, and TP53 pathway alterations. Quantitative genomic metrics,

including nonsynonymous tumor mutation burden, total mutation count, tumor break load, fraction of genome altered, and aneuploidy score, were significantly enriched in MAPK-altered tumors. Hypoxia-related signatures (Winter, Buffa, and Ragnum scores) also demonstrated significant differences between groups. Additionally, TGFβ pathway and SMAD4 alterations were associated with MAPK pathway status in the gemcitabine-treated context. Collectively, these findings reveal that MAPK-altered PDAC treated with gemcitabine exhibits a distinct molecular and genomic instability profile, underscoring the value of conversational AI for rapidly uncovering multidimensional pathway-dependent associations in treatment-specific settings.
